# Camptothecin effectively treats obesity in mice through GDF15 induction

**DOI:** 10.1371/journal.pbio.3001517

**Published:** 2022-02-24

**Authors:** Jun Feng Lu, Meng Qing Zhu, Bao Cai Xie, Xiao Chen Shi, Huan Liu, Rui Xin Zhang, Bo Xia, Jiang Wei Wu

**Affiliations:** Key Laboratory of Animal Genetics, Breeding and Reproduction of Shaanxi Province, College of Animal Science and Technology, Northwest A&F University, Yangling, Shaanxi, China; Columbia University, UNITED STATES

## Abstract

Elevated circulating levels of growth differentiation factor 15 (GDF15) have been shown to reduce food intake and lower body weight through activation of hindbrain receptor glial-derived neurotrophic factor (GDNF) receptor alpha-like (GFRAL) in rodents and nonhuman primates, thus endogenous induction of this peptide holds promise for obesity treatment. Here, through *in silico* drug-screening methods, we found that small molecule Camptothecin (CPT), a previously identified drug with potential antitumor activity, is a GDF15 inducer. Oral CPT administration increases circulating GDF15 levels in diet-induced obese (DIO) mice and genetic *ob/ob* mice, with elevated *Gdf15* expression predominantly in the liver through activation of integrated stress response. In line with GDF15’s anorectic effect, CPT suppresses food intake, thereby reducing body weight, blood glucose, and hepatic fat content in obese mice. Conversely, CPT loses these beneficial effects when *Gdf15* is inhibited by a neutralizing antibody or AAV8-mediated liver-specific knockdown. Similarly, CPT failed to reduce food intake and body weight in GDF15’s specific receptor GFRAL-deficient mice despite high levels of GDF15. Together, these results indicate that CPT is a promising anti-obesity agent through activation of GDF15-GFRAL pathway.

## Abstract 2

Elevated circulating levels of growth differentiation factor 15 (GDF15) have been shown to reduce food intake and lower body weight in rodents and nonhuman primates. This study reveals that the small molecule Camptothecin induces endogenous GDF15, suppressing food intake and reducing body weight in obese mice, suggesting a promising candidate for anti-obesity treatment.

## Introduction

Obesity is a global health problem that predisposes people to diseases such as type 2 diabetes, cardiovascular diseases, fatty liver, and even cancer [1,2]. Therefore, it is a great threat to human health and a heavy burden on public health systems. Due to unsatisfactory results of interventions on dietary and physical activity, the use of weight loss drugs has been proposed as a more effective choice for long-term obesity management. In the past several decades, a plethora of efforts have been devoted to explore the anti-obesity targets and develop weight management agents [3]. Main medications currently approved by FDA for obesity management include orlistat, phentermine, phentermine/topiramate extended release, liraglutide, and semaglutide [4,5]. Some of them have achieved desirable results for a limited population [6]. However, the problem has not been well resolved because (1) many targets have limitations and functions only for a small population; and (2) adverse effects such as gastrointestinal disorders [7] and myopathy [8] have been repeatedly reported in users taken FDA-approved weight control medications. Therefore, exploring new therapeutic targets and understanding their regulations for drug development are current areas of intense interest and active investigation.

Growth differentiation factor 15 (GDF15), also known as macrophage inhibitory cytokine-1, has emerged as a new anti-obesity target [9,10]. It is a stress-responsive cytokine expressed in a variety of tissues and secreted into circulation in response to many stimuli as part of a wide array of disease processes [11]. Elevating GDF15 levels by transgenic overexpression or pharmacological administration in mice and nonhuman primates lead to a marked fall in body weight [12–14]. Mice lacking GDF15 become more obese on a high-fat diet (HFD) than wild-type (WT) controls [15]. Recently, with the discovery of GDF15’s specific receptor glial-derived neurotrophic factor (GDNF) receptor alpha-like (GFRAL) [13,16,17], enthusiasm is heightened in understanding this hormone’s regulation and exploring its therapeutic application in obesity treatment [18]. Thus, the aim of this work was to explore a pharmacological GDF15 inducer and test its anti-obesity efficacy in order to provide alternative anti-obesity therapeutics.

With advances in bioinformatics and network pharmacology, the Connectivity Map (CMAP), a database collecting gene expression profiles of drug-treated human cell lines [19], has been widely used for anti-obesity purpose to screen leptin sensitizer [20,21] and thermogenic activators [22]. In the current work, we screened the CMAP database for small molecules with gene expression signatures of GDF15 and identified Camptothecin (CPT) as a potential GDF15 inducer. CPT is a pentacyclic quinoline alkaloid present in wood, bark, and fruit of the Asian tree *Camptotheca acuminate* [23]. The US National Cancer Institute screening program identified CPT as a drug with potential antitumor activity in 1966, mechanistically through inhibition of type 1 DNA topoisomerase (TOP 1) and induction of dsDNA breaks in cancer cells [24]. Limited Phase II clinical trials of CPT by a single injection every 3 weeks at initial doses ranging between 90 and 180 mg/m^2^ in patients with advanced gastrointestinal adenocarcinoma gained partial encouraging objective responses [25]. However, using these testing doses, frequency, and administration mode, some adverse events including nausea, vomiting, dermatitis, diarrhea, cystitis, leukopenia, thrombocytopenia, and anemia were reported, thus hampering its initial clinical development as a chemotherapeutic drug. Here, by screening of CMAP database, we found a strong correlation between CPT and GDF15 expression, suggesting a potential anti-obesity property of CPT.

In this study, we perform a series of animal experiments to determine the potential link between CPT and the changes in circulating GDF15 levels, food intake, and body weight. We show that 1 mg kg^−1^ of CPT administration induces mild circulating GDF15 elevation, which suppresses food intake and reduces body fat mass without causing adverse effects. Using either GDF15 neutralization or GFRAL deficiency in mice, we show that the weight lowering effects of CPT depend on GDF15-GFRAL axis. We also corroborate that the main source of CPT-induced GDF15 derives from activation of hepatic activating transcription factor 4 (ATF4) and C/EBP homologous protein (CHOP). These findings suggest that CPT could be a potential anti-obesity agent when applied at a once daily dose of 1 mg kg^−1^ of body weight orally.

## Results

### Screening of CMAP database identifies CPT as a potential GDF15 inducer

**Fig 1:**
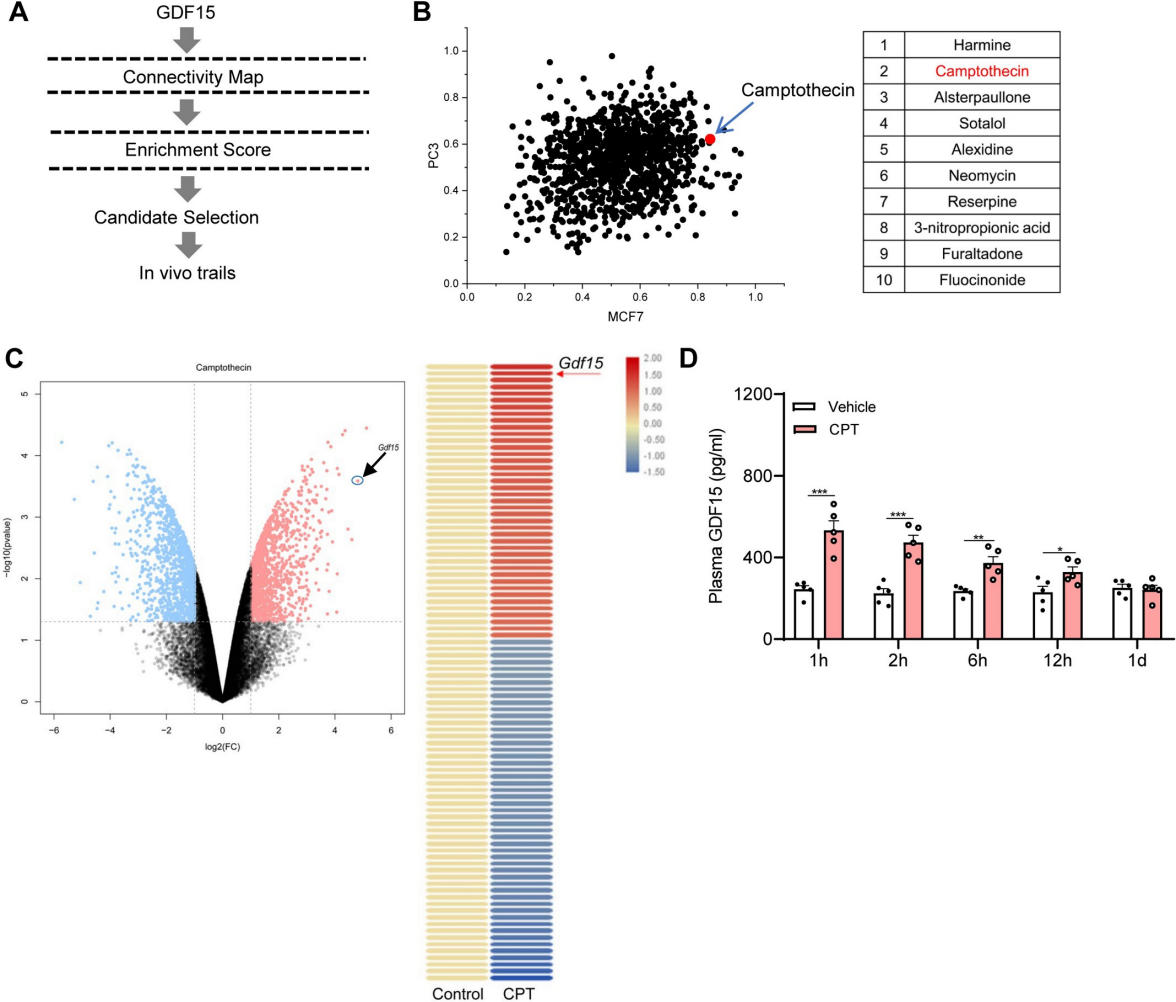
Identification of CPT as a potential GDF15 inducer. **(A)** Schematic diagram depicting the workflow of obtaining gene expression signatures from the CMAP database to identify GDF15 inducer. **(B)** Left panel: the dot distribution of enrichment scores of the PC3 and MCF7 cell line. The expression levels of *Gdf15* induced by different small molecules range from 0 to 1 enrichment score. In this study, we choose a list of small molecules with an enrichment score over 0.6. Each point represents an individual small molecule. The red dot indicated by the arrow represents CPT. Right panel: top 10 small molecules. **(C)** Left panel: scatter plot showing the Log_2_ fold changes of gene abundance upon 2 μM CPT treatment in 293T cell line. Differentially abundant genes (*n =* 828, *P* < 0.05) are indicated [red, up-regulated (*n* = 398) and blue down-regulated (*n* = 430)]. The circled red dot indicated by the arrow represents *Gdf15*. Significantly abundant genes were evaluated by Student *t* test (FDR = 0.05). Right panel: heatmap showing the top 50 up-regulated (red) and down-regulated (blue) genes obtained from 293T cells treated with vehicle or 2 μM CPT. **(D)** Plasma levels of GDF15 measured 1 h, 2 h, 6 h, 12 h, or 24 h in DIO mice receiving a single oral dose of 1 mg kg^−1^ CPT, * *P* < 0.05, ** *P* < 0.01, *** *P* < 0.001. *n =* 5 per group. The underlying data for this figure can be found in **S1 Data**. CMAP, Connectivity Map; CPT, Camptothecin; FDR, false discovery rate; GDF15, growth differentiation factor 15.

GDF15 has become a keen target of interest for anti-obesity therapies [26]. Screening of small molecules that could induce GDF15 expression in vivo holds promise for clinical applications. For this purpose, we used CMAP, which comprises a database of whole-genome gene expression profiles derived from human cell lines treated with more than 6,000 small molecules [19], to screen for small molecules that could elevate the expression of *Gdf15* (Fig 1A). A list of 109 CMAP molecules was selected by analyzing signatures of the cell line MCF7 and PC3. The distribution of individual small molecules in the selected library is shown based on their enrichment scores (S1 Fig). Combined distribution of the enrichment scores of the selected molecules for both MCF7 and PC3 ranked CPT as the second top compound in the database for *Gdf15* expression (the first compound has been prohibited by Therapeutic Goods Administration) (Fig 1B). Of note, an FDA-approved CPT analog Irinotecan (CPT-11) is included in the CMAP database. Nonetheless, its enrichment scores rank far below 100 in terms of *Gdf15* expression. Analysis of the expression profile of CPT-treated 293T cell line from the available GEO dataset (GSE2451) revealed *Gdf15* as the second up-regulated gene among the top 50 differentially expressed genes as shown by volcano analysis (Fig 1C, left) and heatmap (Fig 1C, right), exhibiting more than 600-fold increase compared with controls. Together, these results suggest that CPT is a potential GDF15 inducer.

### CPT induces GDF15 secretion in mice

To test the effect of CPT on GDF15 induction in vivo, both HFD-induced obese mice (DIO) and genetic *ob/ob* mice were orally treated at an acute single dose of either vehicle or CPT (1 mg kg^−1^ of body weight). The human equivalent dose (HED) of CPT used in mice were 3.01 mg/m^2^ for a 60-kg person based on body surface area normalization [27], which were approximately 30- to 60-fold lower than those tested in Phase II clinical trials for advanced gastrointestinal adenocarcinoma patients (90 to 180 mg/m^2^). The plasma concentration time profile for 1 mg kg^−1^ of CPT in DIO mice was shown in S2 Fig. CPT concentrations were approximately 9.5 ng ml^−1^ at 1 h, and then decreased to 1 ng ml^−1^ at 12 h. Consistent with the above screening results, 1 mg kg^−1^ of CPT administration resulted in a 2.2- and 2.0-fold increase in circulating GDF15 levels after 1 h in DIO mice (Fig 1D) and *ob/ob* mice (S3 Fig), respectively. Of note, we (S4 Fig) and others [28] showed that CPT-11 was unable to induce GDF15 secretion in mice. These data support that CPT induces circulating GDF15 secretion in obese mice.

### CPT ameliorates obesity and associated metabolic abnormalities in mice

**Fig 2:**
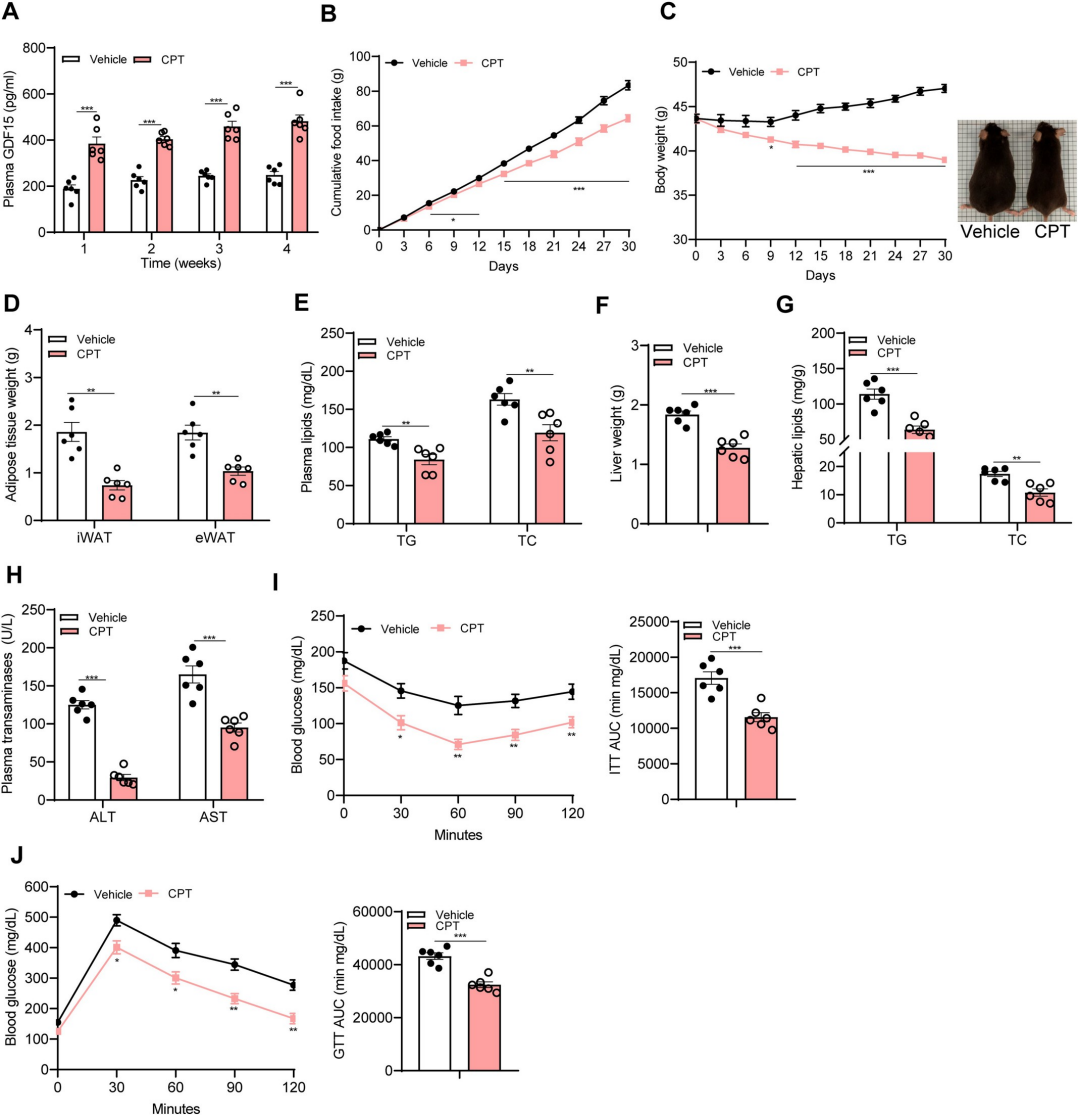
CPT reduces body weight and food intake in DIO mice accompanied by elevated circulating GDF15 levels. **(A-J) Animal protocol 1**: DIO mice orally received vehicle or CPT (1 mg kg^−1^ day^−1^) for 30 days. **(A)** Plasma levels of GDF15 at indicated time points. **(B)** Cumulative food intake. **(C)** Consecutive body weight. **(D)** Tissue weights of iWAT and eWAT in CPT-treated mice and corresponding controls. **(E)** Plasma levels of TG and TC. **(F)** Liver weight. **(G)** Hepatic TG and TC contents. **(H)** Plasma levels of ALT and AST. **(I)** ITT and AUC of ITT (day 15). **(J)** GTT and AUC of GTT (day 21). Data are presented as mean ± SEM. * *P* < 0.05, ** *P* < 0.01, *** *P* < 0.001, *n =* 6 per group. The underlying data for this figure can be found in **S1 Data**. AUC, area under the curve; ALT, alanine aminotransferase; AST, aspartate aminotransferase; CPT, Camptothecin; DIO, diet-induced obese; eWAT, epididymal white adipose tissue; GDF15, growth differentiation factor 15; GTT, glucose tolerance test; ITT, insulin tolerance test; iWAT, inguinal white adipose tissue; TC, total cholesterol; TG, triglyceride.

To determine whether long-term CPT treatment could sustain high circulating levels of GDF15 and thereby alleviates obesity in mice, we performed longitudinal study in which both DIO mice and *ob/ob* mice orally received 1 mg kg^−1^ day^−1^ of CPT for 30 days. We observed significantly increased circulating levels of GDF15 in CPT-treated DIO mice (1.6- to 1.9- fold, Fig 2A) and *ob*/*ob* mice (1.6- to 1.8- fold, S5A Fig) at the 4 indicated time points (1 w, 2 w, 3 w, and 4 w). In line with this, CPT treatment reduced accumulative food intake in DIO mice starting from day 6 (12.01%) till the end of the observation period (Fig 2B), and in *ob*/*ob* mice starting from day 9 (S5B Fig). CPT treatment reduced the body weight of DIO mice from 43.63 ± 0.54 g to 39.00 ± 0.20 g (on day 1 versus day 30, *P* < 0.001) (Fig 2C), equivalent to a weight loss of 10.58% ± 0.91% relative to their initial weights. The body weight of vehicle-treated control mice increased by 3.39 g ± 0.53 g, corresponding to 8.25% ± 1.32% weight gain relative to their initial weights. Body weight reduction was also shown in CPT-treated *ob/ob* mice (6.03% ± 0.34% of weight loss) (S5C Fig). In contrast to obese mice, a 30-day testing of 1 mg kg^−1^ day^−1^ of CPT in lean mice showed similar food intake (S6A Fig) and body weight (S6B Fig) to their vehicle controls. The nonelevated GDF15 levels (S6C Fig) might be a main cause for absence of impact on body weight in CPT-treated lean mice.

The weight loss in CPT-treated obese mice was mainly attributed to the reductions in fat mass: 60.23% in inguinal white adipose tissue (iWAT) and 43.85% in epididymal white adipose tissue (eWAT) (Fig 2D) of DIO mice. Fat reductions were also shown in CPT-treated *ob*/*ob* mice (37.12% in iWAT and 19.83% in eWAT) (S7A Fig). Consistent with these data, CPT markedly reduced plasma levels of triglyceride (TG) and total cholesterol (TC) in both DIO mice and *ob/ob* mice (Fig 2E, S7B Fig). Additionally, CPT-treated obese mice showed reduced hepatic lipid content and liver weight (Fig 2F and 2G, S7C and S7D Fig), accompanied with reductions in levels of alanine aminotransferase (ALT) and aspartate aminotransferase (AST) (Fig 2H, S7E Fig), suggesting an improvement in obesity-associated fatty liver and liver damage upon CPT treatment. There was no difference in the weights of kidney and spleen between CPT-treated obese mice and vehicle controls (S7F Fig). Since high circulating GDF15 was positively correlated with cachexia, characterized by great loss of adipose tissue and skeletal muscle (sarcopenia), in some types of cancer patients [29,30], we examined whether CPT-induced GDF15 elevation causes sarcopenia in the context of obesity in our model. Measurements of skeletal muscle mass showed similar weights of [gastrocnemius (GAS), quadriceps (QUA), soleus (SOL), and extensor digitorum longus (EDL)] between CPT-treated *ob*/*ob* mice and vehicle controls (S7G and S7H Fig), as was the case in CPT-treated DIO mice and controls (S8A–S8C Fig). We even observed improvement in muscle grip strength (grip experience: 1.59-fold; grip strength: 1.39-fold; and swimming time: 1.58-fold) in CPT-treated DIO mice (S9A–S9C Fig), arguing for normal or even improved skeletal muscle function.

The metabolic benefits of CPT on glucose homeostasis were revealed by glucose tolerance test (GTT) and insulin tolerance test (ITT). CPT-treated DIO mice showed improved responsiveness to insulin stimulation than the corresponding controls (Fig 2I), suggesting improved insulin sensitivity. Similarly, we observed faster glucose disposal capacity in CPT-treated DIO mice than in controls (Fig 2J), indicating enhanced glucose tolerance upon CPT treatment. CPT treatment improved glucose tolerance (S10A Fig) of *ob*/*ob* mice without altering their insulin sensitivity (S10B Fig). We next asked whether CPT itself is improving glucose metabolism or this effect is attributed to CPT-induced weight loss. To test it, we undertook further GTT in a cohort of weight-matched DIO mice receiving a single dose of CPT (1 mg kg^−1^). In these mice, CPT had no significant effect upon glucose disposal (S11 Fig). Thus, the beneficial effect of CPT on glucose metabolism is a secondary response to weight loss. Collectively, these results demonstrate that CPT ameliorates obesity, thereby reducing hyperlipidemia, fatty liver, and hyperglycemia in obese mice.

Given the documented adverse effects of CPT in clinical trials, we carefully examined most of these aspects in mice at the end of the 30-day treatment. CPT-treated mice showed similar counts of leucocyte and platelet, and comparable levels of hemoglobin to their vehicle controls (S1 Table), indicating absence of leukopenia, thrombocytopenia, and anemia. We did not observe abnormalities in hair, skin, mouth, and bladder in CPT-treated mice (S1 Table), indicating absence of alopecia, dermatitis, stomatitis, and cystitis. Moreover, CPT-treated mice showed similar levels of creatinine (CREA) and blood urea nitrogen (BUN) to their controls (S12A–S12D Fig). These results suggest that oral CPT (1 mg kg^−1^) does not cause apparent adverse effects in mice. Together, our results demonstrate that CPT ameliorates obesity and associated metabolic abnormalities in mice, which may be due to elevations of circulating GDF15.

### The anti-obesity effect of CPT in mice is achieved by appetite suppression without affecting energy expenditure

To test whether reduced food intake solely or along with altered energy expenditure (EE) contributes to the body weight control effect of CPT in mice, new cohorts of DIO mice treated with either CPT or vehicle were raised. We first applied a 9-day pair-feeding regime to mice to investigate whether this could abrogate the weight difference between CPT-treated mice and controls. In this experiment, the average daily food intake of CPT-treated mice was the same as that of the pair-fed vehicle groups. We observed similar body weight in CPT-treated mice to their pair-fed vehicle controls (S13A–S13C Fig), suggesting that food suppression is the main contributing factor responsible for body weight loss. Furthermore, we undertook indirect calorimetry in CPT-treated and vehicle control mice under ad libitum to establish whether there are additional effects on EE. CPT-treated mice showed comparable EE to controls when data were analyzed by analysis of covariance (ANCOVA) with body weight as the covariate (S14A–S14C Fig), as were of body temperature and heat production (S14D and S14E Fig). Collectively, these results show that the anti-obesity effect of CPT is due to the suppression of food intake, which is consistent with the reported anorectic effects of GDF15.

Given that exogenous injection of GDF15 in mice was reported to produce food aversion and visceral malaise under certain circumstances [31], we tested whether CPT-induced endogenous GDF15 elevation and food intake suppression could be of relevance to these abnormal behaviors using conditioned taste aversion (CTA) test in mice and pica test in rats as described [31–33]. Despite high levels of GDF15, CPT-treated mice did not show obvious food avoidance (S15A–S15C Fig). In the pica test, CPT-treated rats showed similar kaolin intake to vehicle control rats for the entire testing period (CPT: 0.53 ± 0.17 g kaolin per day; vehicle: 0.33 ± 0.12 g) (S16A and S16B Fig), suggesting absence of sickness behaviors indicative of nausea or malaise upon low-dose CPT (1 mg kg^−1^ CPT) treatment. The mean daily food intake was lower in CPT-treated rats than in vehicle controls (CPT: 20.01 ± 0.58 g; vehicle: 22.14 ± 0.64 g per day; *P* < 0.05) (S16C and S16D Fig). At the end of the experiment, CPT-treated rats showed pronounced reductions in body weight gain compared to vehicle controls (CPT: −6.53 ± 1.57 g; vehicle: 24.82 ± 3.18 g; *P* < 0.01) (S16E Fig). Together, these results indicate that (1) 1 mg kg^−1^ day^−1^ of CPT-induced GDF15 is not sufficient to cause food aversion and visceral malaise; and/or (2) the effects of endogenous GDF15 might be different from that of exogenous.

### Antibody neutralization of GDF15 abolishes the beneficial metabolic effects of CPT in obese mice

**Fig 3:**
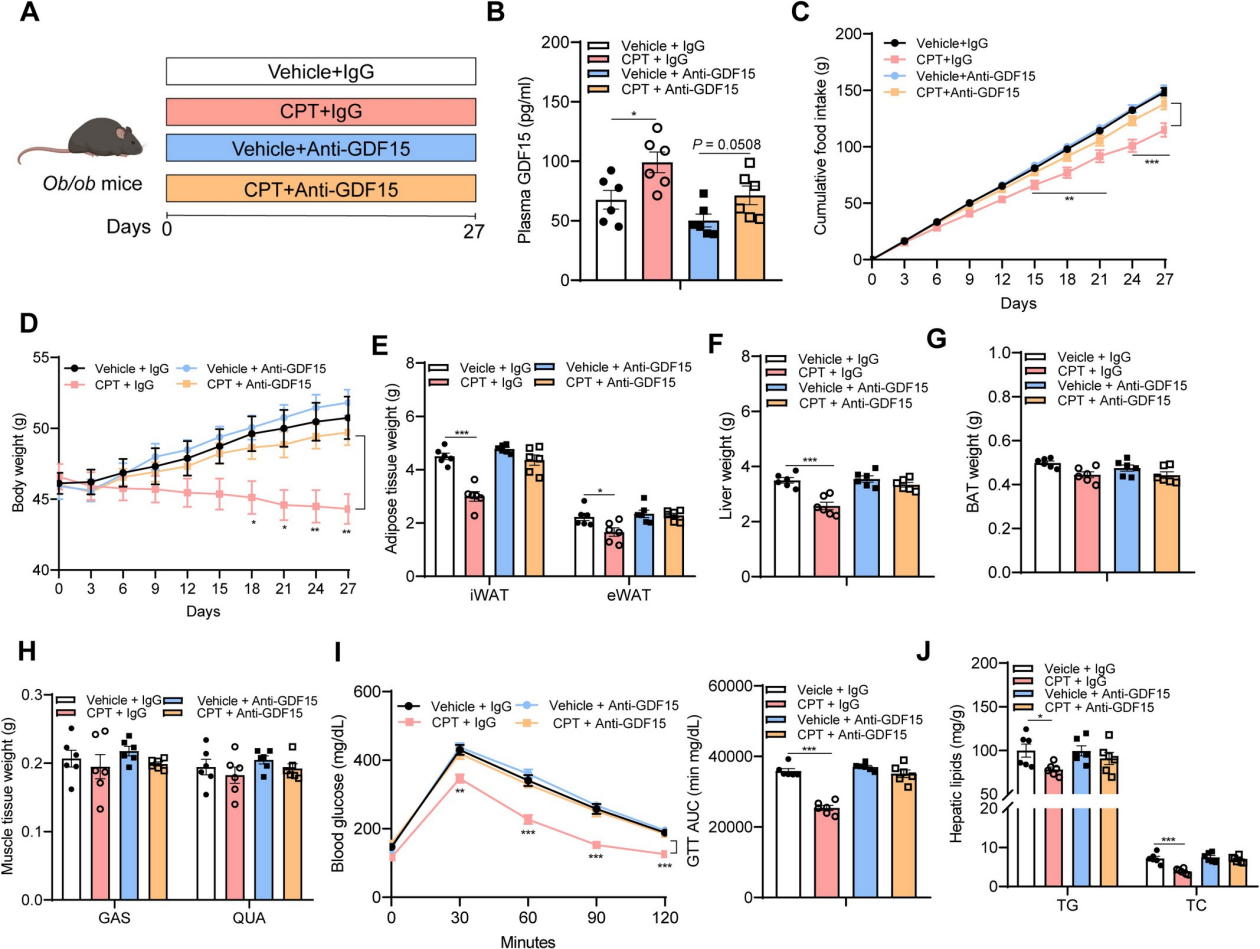
GDF15 neutralization blocks the effects of CPT to suppress food intake and body weight in *ob/ob* mice. **(A-J) Animal protocol 2**: **(A)** *Ob/ob* mice were treated with either 5 mg kg^−1^ GDF15-neutralizing antibody or control IgG every other day for 27 days through subcutaneous injection, 24 h before oral CPT (1 mg kg^−1^ day^−1^) or vehicle. Created with biorender.com. **(B)** Effect of CPT on circulating levels of GDF15 after 21 days of treatment. **(C)** Cumulative food intake. **(D)** Consecutive body weight of mice. **(E)** Adipose tissue weights. **(F)** Liver weight. **(G)** BAT weight. **(H)** Mass of GAS and QUA. **(I)** GTT and AUC of GTT (day 18). **(J)** Hepatic TG and TC contents. Data are presented as mean ± SEM. * *P* < 0.05, ** *P* < 0.01, *** *P* < 0.001, *n =* 6 per group. The underlying data for this figure can be found in **S1 Data**. AUC, area under the curve; BAT, brown adipose tissue; CPT, Camptothecin; eWAT, epididymal white adipose tissue; GAS, gastrocnemius; GDF15, growth differentiation factor 15; GTT, glucose tolerance test; IgG, immunoglobulin G; iWAT, inguinal white adipose tissue; QUA, quadriceps; TC, total cholesterol; TG, triglyceride.

CPT treatment elevates circulating GDF15 in mice, to further test whether GDF15 is indispensable for CPT-mediated metabolic benefits, the action of GDF15 was inhibited by a GDF15-neutralizing antibody (Fig 3A and 3B). The efficiency of this antibody was validated in a cohort of WT mice received either exogenous GDF15 alone or along with coadministration of this neutralizing antibody (S17A and S17B Fig). CPT-consuming *ob/ob* mice treated with anti-GDF15 showed similar amount of food intake to vehicle control mice treated with anti-GDF15, whereas the food intake reduction seen in CPT-consuming *ob/ob* mice treated with immunoglobulin G (IgG) was maintained (Fig 3C), suggesting that GDF15 directly mediates the anorectic effect of CPT. Consistent with this, the body weight gain in CPT-treated mice (3.68 ± 0.62 g, 8.02% ± 1.35%) was not different from that of vehicle controls (5.90 ± 0.60 g, 12.91% ± 1.41% of body weight) upon GDF15 neutralization. In contrast, the weight loss seen in CPT-treated mice was preserved with IgG treatment compared with their corresponding controls, reaching 4.90% ± 0.68% below starting body weight (Fig 3D). The significant reductions in fat mass and liver weight seen with CPT treatment were not observed in the anti-GDF15 group (Fig 3E and 3F). The weights of brown adipose tissue (BAT), GAS, and QUA were not altered by GDF15 neutralization (Fig 3G and 3H). Moreover, GDF15 neutralization abrogated the beneficial effects of CPT on glucose homeostasis (Fig 3I) and prevention of hepatic steatosis (Fig 3J). Together, these results indicate that GDF15 mediates the beneficial metabolic effects of CPT in mice.

### CPT-induced GDF15 production originates from the liver through activation of ISR pathway

**Fig 4:**
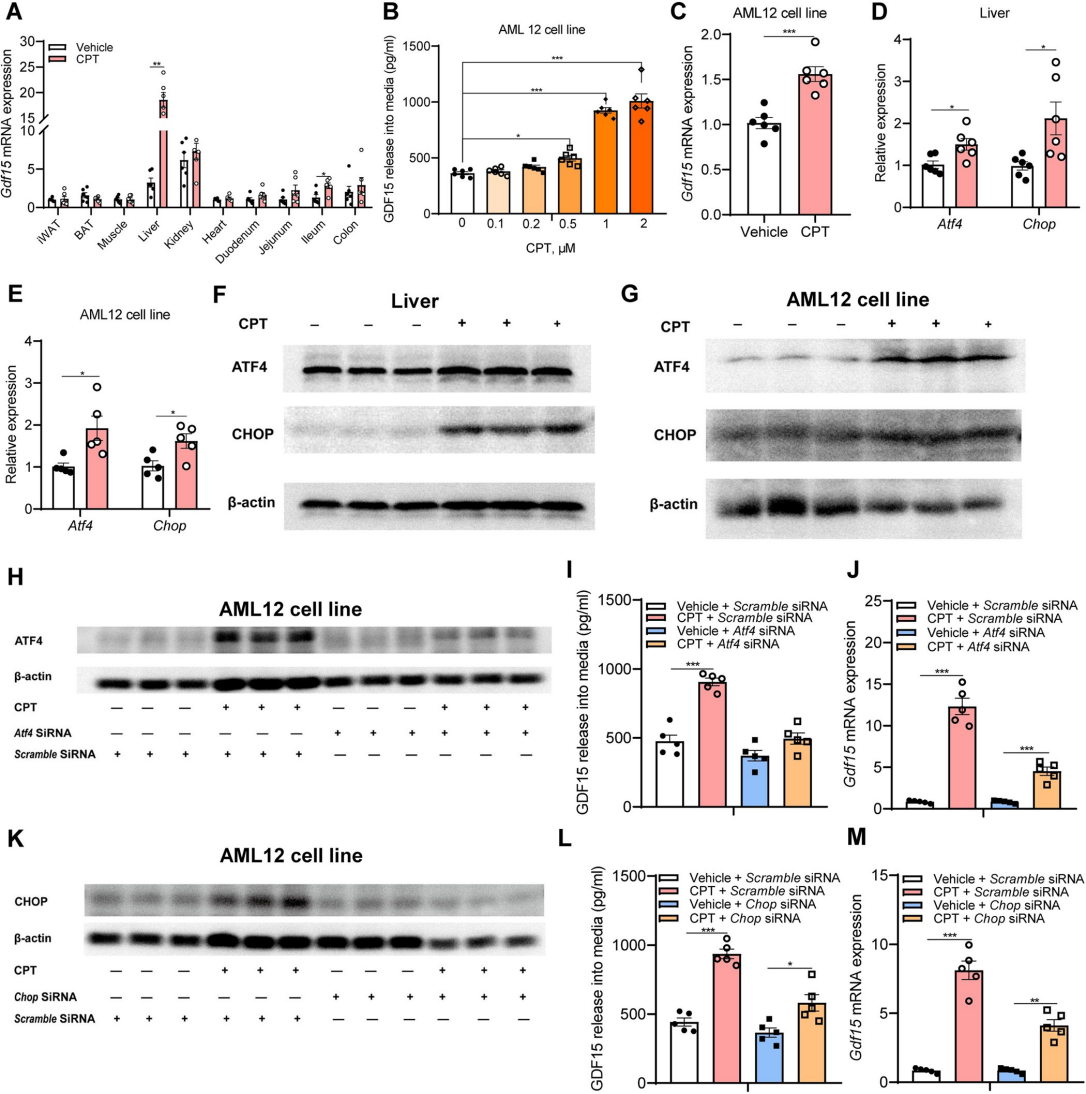
CPT increases GDF15 release from hepatocytes through activation of ISR pathway. **(A)** *Gdf15* mRNA expression in iWAT, BAT, skeletal muscle, liver, kidney, and gut of DIO mice after 30 days of treatment (*n =* 6). **(B)** GDF15 release in medium of cultured hepatocytes (AML12) treated with indicated doses of CPT for 24 h (*n* = 6). **(C)** The induction of *Gdf15* mRNA expression by 1 μM CPT treatment for 24 h in AML 12 cells (*n* = 6). **(D)** mRNA expression levels of *Atf4* and *Chop* in livers of DIO mice after 30 days of treatment (*n* = 6). **(E)** mRNA expression levels of *Atf4* and *Chop* in 1 μM CPT-treated AML12 cells for 24 h (*n* = 5). **(F-G)** Immunoblot analysis of ATF4 and CHOP relative to β-actin in livers of DIO mice treated with 1 mg kg^−1^ of CPT for 30 days and in AML 12 cells treated with 1 μM CPT for 24 h (*n* = 3), respectively. **(H)** siRNA knockdown of *Atf4* blunts 1 μM CPT-induced ATF4 expression (*n* = 3). **(I-J)** GDF15 release and relative *Gdf15* expression in control siRNA and *Atf4* siRNA transfected AML 12 cells treated with 1 μM CPT for 24 h (*n* = 5). **(K)** siRNA knockdown of CHOP blunts CPT-induced CHOP expression (*n* = 3), **(L-M)** GDF15 release and relative *Gdf15* expression in control siRNA and *Chop* siRNA transfected AML 12 cells treated with 1 μM CPT for 24 h (*n* = 5). Data are presented as mean ± SEM. * *P* < 0.05, ** *P* < 0.01, *** *P* < 0.001. The underlying data for this figure can be found in **S1 Data**. The original blot for this figure can be found in **S1 Raw Image**. ATF4, activating transcription factor 4; BAT, brown adipose tissue; CHOP, C/EBP homologous protein; CPT, Camptothecin; DIO, diet-induced obese; GDF15, growth differentiation factor 15; ISR, integrated stress response; iWAT, inguinal white adipose tissue.

GDF15 is a circulating protein that could be potentially produced by multiple tissues under different conditions [34,35]. To clarify which organs of CPT-mediated GDF15 production were mainly derived from, we examined *Gdf15* gene expression in a tissue panel including iWAT, BAT, skeletal muscle, liver, kidney, heart, and gut, which have been reported as potential sources of circulating GDF15 [36], obtained from DIO mice treated with either CPT or vehicle for 30 days. Compared with controls, markedly increased *Gdf15* mRNA expression was observed in CPT-treated livers (Fig 4A). To further determine whether hepatocytes are capable of responding to CPT with an increase in GDF15, we incubated mouse hepatocytes (AML12) with CPT and found induction of GDF15 release into the medium in a dose-dependent manner (Fig 4B) and a clear elevation of *Gdf15* mRNA expression in hepatocytes (Fig 4C), demonstrating a direct link between CPT treatment and hepatic GDF15 secretion.

Based on previous work that GDF15 is a downstream target of the cellular integrated stress response (ISR) pathway [37], we hypothesized that ATF4 and CHOP, key transcriptional regulators of the ISR, might be involved in this process. In agreement with this, we found markedly increased mRNA and protein expression of ATF4 and CHOP in CPT-treated liver and hepatocytes (Fig 4D–4G). Conversely, either siRNA-mediated knockdown of *Atf4* or *Chop* in hepatocytes blunted CPT-induced *Gdf15* mRNA expression and GDF15 release into the medium (Fig 4H–4M), suggesting an essential role of ISR pathway in CPT-induced hepatic GDF15 production. To examine whether CPT-induced ISR activation is specific to liver, we measured protein expression levels of ATF4 and CHOP in ileum due to its high *Gdf15* mRNA expression upon CPT treatment. Unlike in liver, their levels in ileum were similar in the 2 groups of mice (S18 Fig). These data indicate that CPT induces hepatic GDF15 production through activation of ISR pathway.

Since CPT is known to be a specific inhibitor of type 1 DNA topoisomerase (TOP 1) and used to induce dsDNA breaks and cancer cell death [38], we investigated whether potential dsDNA breaks could account for CPT-induced ISR activation in liver. To this end, we measured the levels of γH2AX, a well-established biomarker of dsDNA breaks [39,40], using an ELISA-based γH2AX pharmacodynamic assay in response to CPT treatment in mouse livers, murine hepatocytes (AML12), and hepatoma cells (Hepa1-6). Similar γH2AX levels were shown in CPT-treated mouse livers and their controls (S19A Fig), as well as in 1 μM CPT-treated AML12 hepatocytes and the DMSO-treated controls. However, 1 μM CPT-treated Hepa1-6 cells exhibited higher γH2AX levels than DMSO-treated controls, indicating increased dsDNA breaks (S19B Fig). These results indicate that dsDNA breaks do not occur in CPT-treated normal livers and thus are not a contributor of CPT-induced hepatic ISR activation and GDF15 induction.

**Fig 5:**
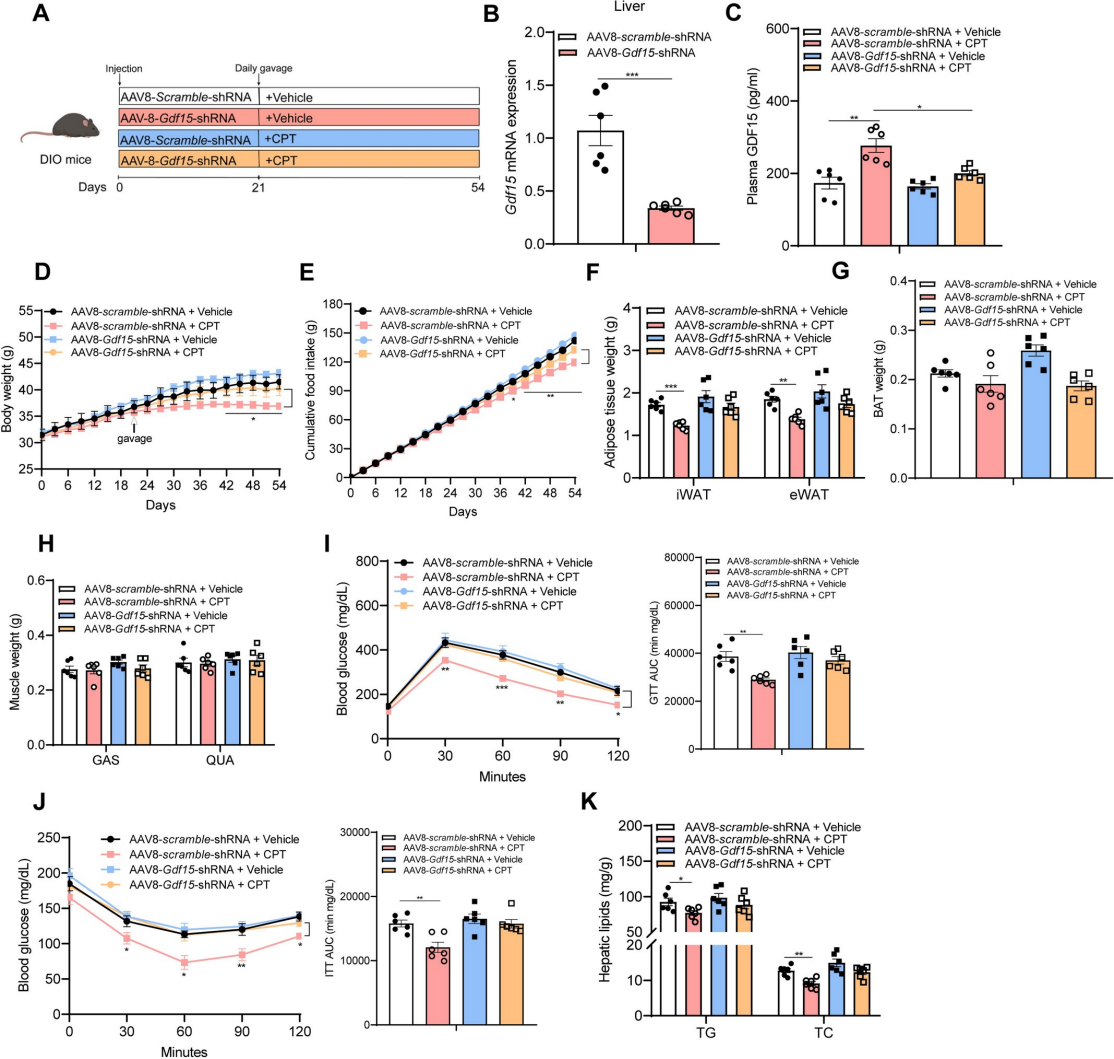
CPT-induced GDF15 expression derives mainly from the liver. **(A-K) Animal protocol 3**: **(A)** Schematic diagram of mice treatment. Created with biorender.com. DIO mice were intravenously injected once with adenovirus (AAV8-*scramble*-shRNA or AAV8-*Gdf15*-shRNA) through the tail vein (1 × 10^11^ vector genomes/mouse). After 21 days, mice were treated daily with either vehicle or CPT for 33 days. Thus, 4 groups of mice (vehicle + AAV8-*scramble*-shRNA, vehicle + AAV8-*Gdf15*-shRNA, CPT + AAV8-*scramble*-shRNA, and CPT + AAV8-*Gdf15*-shRNA) were studied here. **(B)** Hepatic *Gdf15* mRNA expression and **(C)** plasma levels of GDF15 after 33 days of treatment. **(D)** Consecutive body weight. **(E)** Cumulative food intake. **(F-G)** Adipose tissue weight. **(H)** Mass of GAS and QUA. **(I)** GTT was performed after 42 days of treatment and AUC of GTT. **(J)** ITT was performed after 49 days of treatment and AUC of ITT. **(K)** Hepatic TG and TC contents. Data are presented as mean ± SEM. * *P* < 0.05, ** *P* < 0.01, *** *P* < 0.001. *n =* 6 per group. The underlying data for this figure can be found in **S1 Data**. AUC, area under the curve; BAT, brown adipose tissue; CPT, Camptothecin; DIO, diet-induced obese; GAS, gastrocnemius; GDF15, growth differentiation factor 15; GTT, glucose tolerance test; ITT, insulin tolerance test; QUA, quadriceps; TC, total cholesterol; TG, triglyceride.

To investigate whether CPT induces hepatic GDF15 production in vivo, we generated mice with liver-specific *Gdf15* knockdown by using AAV8 system carrying a *Gdf15* shRNA as illustrated in Fig 5A. As predicted, CPT-induced hepatic *Gdf15* mRNA expression and secretion in DIO mice were drastically reduced when receiving AAV8-*Gdf15* shRNA (Fig 5B and 5C) in contrast to scramble shRNA. Accordingly, the suppression of food intake and the subsequent body weight loss by CPT treatment were reduced upon hepatic *Gdf15* knockdown (Fig 5D and 5E). The fat-reducing effect of CPT in DIO mice was markedly diminished with AAV-*Gdf15* treatment (Fig 5F), with no alterations in the weights of BAT (Fig 5G) and skeletal muscle (GAS and QUA) (Fig 5H). Moreover, the beneficial effects of CPT on glucose homeostasis and insulin sensitivity disappeared upon hepatic *Gdf15* knockdown, showing comparable levels of glucose-mediated whole-body glucose disposal as well as insulin-stimulated glucose uptake in CPT-treated mice and vehicle controls (Fig 5I and 5J). CPT induced reductions in hepatic TG and TC were markedly diminished in DIO mice with AAV-*Gdf15* treatment (Fig 5K). We, therefore, conclude that the liver is the main site for CPT-induced GDF15 production.

### GFRAL is the downstream effector of CPT and the GDF15-GFRAL axis mediates the beneficial actions of CPT in mice

**Fig 6:**
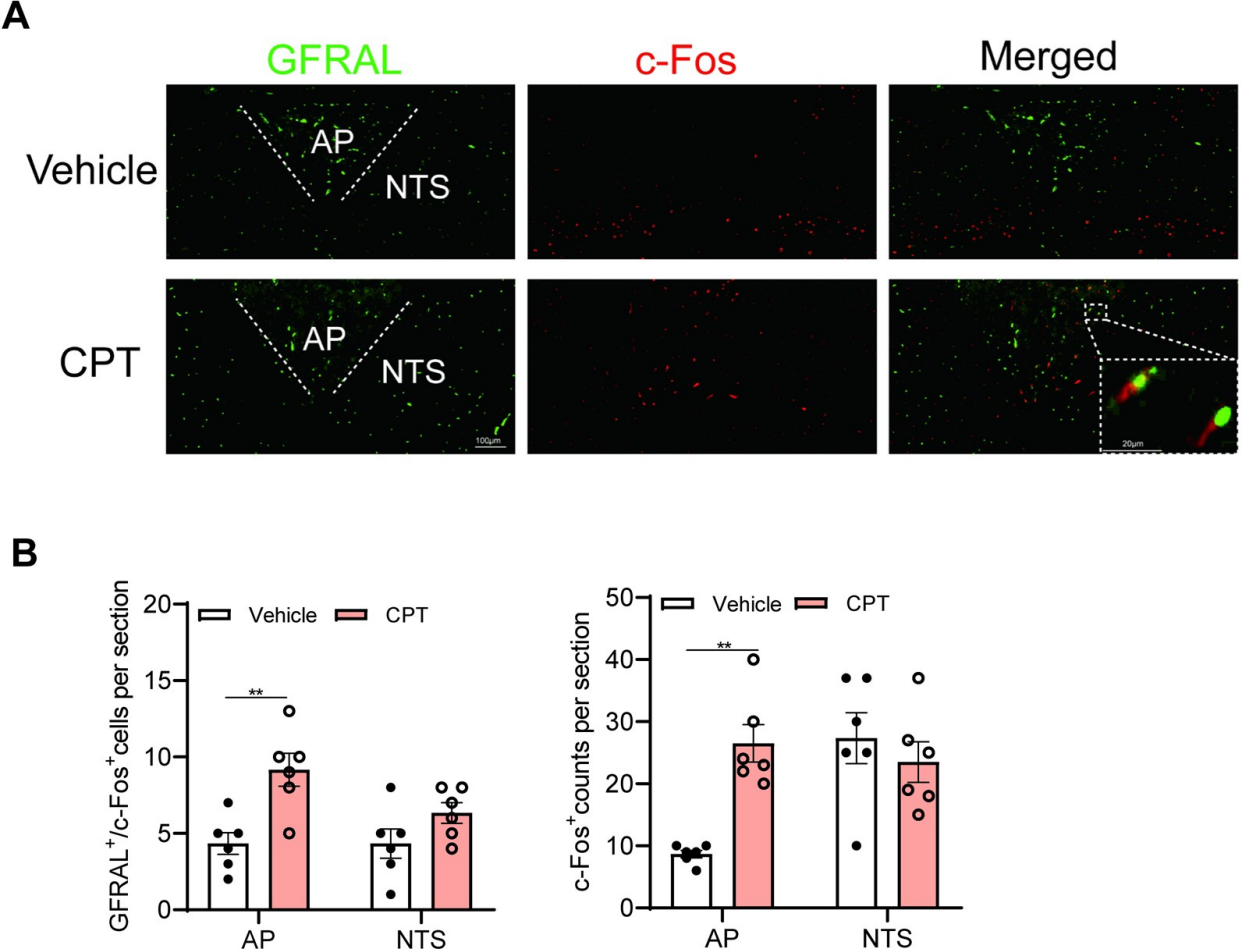
Neuronal activation in CPT-treated mice. DIO mice (weighing 40 to 42 g) received vehicle or CPT (1 mg kg^−1^); 60 min later, brains were harvested, and tissue sections were subjected to immunofluorescence staining with anti-c-Fos (red) and anti-GFRAL (green). **(A)** Representative immunofluorescence image of the AP/NTS in CPT- or vehicle-treated DIO mice showing the distribution of GFRAL neurons and the colocalization with the marker of neuronal activation c-Fos. **(B)** Quantification of CPT-induced c-Fos expression in the AP/NTS (*n =* 6 per group). Data are presented as mean ± SEM. * *P* < 0.05, ** *P* < 0.01, *** *P* < 0.001. The underlying data for this figure can be found in **S1 Data**. AP, area postrema; CPT, Camptothecin; DIO, diet-induced obese; GFRAL, GDNF receptor alpha-like; NTS, nucleus tractus solitarius.

**Fig 7:**
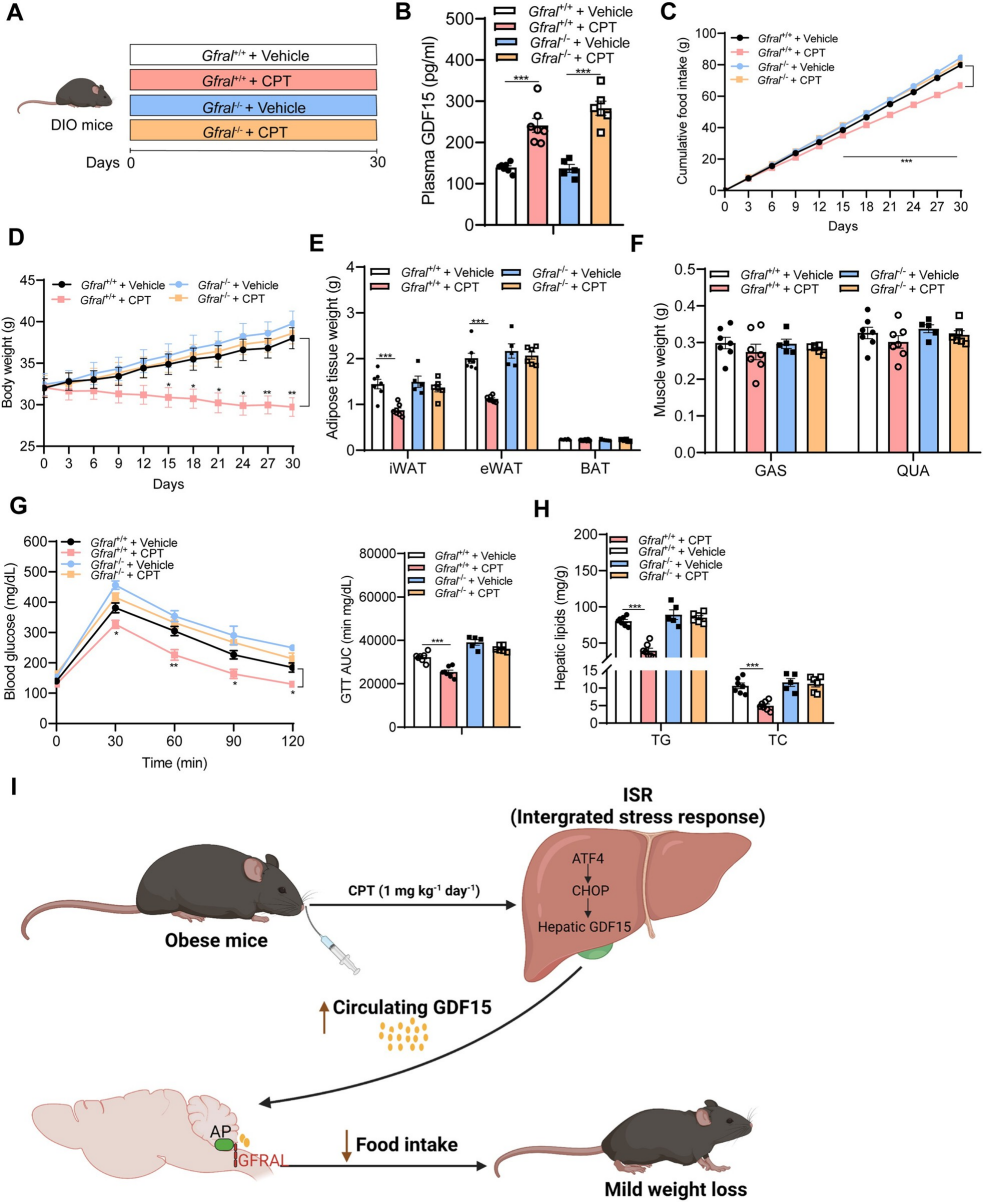
GDF15/GFRAL pathway is required for the weight loss effects of CPT in obese mice. **(A-H) Animal protocol 4**: **(A)** Schematic diagram of mice treatment. WT and *Gfral*^−/−^ mice were subjected to oral administration of vehicle or CPT (1 mg kg^−1^) for 30 days. Created with biorender.com. **(B)** Plasma levels of GDF15 (day 27). **(C)** Cumulative food intake. **(D)** Body weight in response to CPT treatment in WT and *Gfral*^−/−^ mice. **(E-F)** Weights of adipose tissue and skeletal muscle. **(G)** GTT and AUC of GTT (day 18). *Gfral*^+/+^ (vehicle, *n* = 7), *Gfral*^+/+^ (CPT, *n* = 7), *Gfral*^−/−^ (vehicle, *n* = 5), *Gfral*^−/−^ (CPT, *n* = 6). **(H)** Hepatic TG and TC contents at day 30. Data are presented as mean ± SEM. * *P* < 0.05, ** *P* < 0.01, *** *P* < 0.001. The underlying data for this figure can be found in **S1 Data**. **(I)** Proposed model for the anti-obesity effects of CPT in obese mice. Created with biorender.com. CPT elevates circulating GDF15 via activation of hepatic ISR pathway. This activates the GDF15 receptor GFRAL in the hindbrain AP, which subsequently suppresses food intake and reduces body weight in obese mice. AP, area postrema; ATF4, activating transcription factor 4; AUC, area under the curve; BAT, brown adipose tissue; CHOP, C/EBP homologous protein; CPT, Camptothecin; DIO, diet-induced obese; eWAT, epididymal white adipose tissue; GAS, gastrocnemius; GDF15, growth differentiation factor 15; GFRAL, GDNF receptor alpha-like; GTT, glucose tolerance test; ISR, integrated stress response; iWAT, inguinal white adipose tissue; QUA, quadriceps; TC, total cholesterol; TG, triglyceride; WT, wild-type.

Having shown that CPT exerts beneficial metabolic actions through induction of hepatic *Gdf15* expression, we next try to identify the downstream effectors of CPT by determining the expression of GDF15 receptor GFRAL. GFRAL is shown to be exclusively expressed in the area postrema (AP) and nucleus tractus solitarius (NTS) [36], and administration of recombinant GDF15 is known to rapidly activate GFRAL-positive neurons in these regions [41]. Immunofluorescence staining of hindbrain sections containing AP and NTS with antibodies against GFRAL and c-Fos showed robust double staining in AP of CPT-treated mice than in vehicle controls, with similar extent of staining in NTS of both mice (Fig 6A and 6B), suggesting that CPT induces the release of GDF15 and activates GFRAL-expressing neurons in AP of mice. To further test whether GFRAL is indispensable for the beneficial effects of CPT, GFRAL-deficient mice (*Gfral*^−/−^) were used (Fig 7A). Although CPT treatment elevated plasma GDF15 levels in *Gfral*^−/−^ mice as in *Gfral*^+/+^ controls (Fig 7B), it failed to reduce food intake, body weight, and fat mass in *Gfral*^−/−^ mice (Fig 7C–7E). The muscle weights (GAS and QUA) were not significantly different among the 4 groups (Fig 7F). Also, the beneficial effects of CPT on glucose tolerance (Fig 7G) and fatty liver prevention (Fig 7H) were not preserved in *Gfral*^−/−^ mice. Together, these results indicate that GFRAL is the downstream effector of CPT and the GDF15-GFRAL axis mediates the beneficial metabolic actions of CPT in mice.

## Discussion

In this study, by screening of CMAP database, we discover that the small molecule CPT acts as an inducer of GDF15. In vivo testing show that CPT induces hepatic expression and secretion of GDF15 in mice, which reduces food intake and thereby normalizing body weight and reversing metabolic dysfunctions in obese mice (Fig 7I). In contrast, antibody neutralization of GDF15 or deficiency of GFRAL in mice abolishes the metabolic benefits of CPT, corroborating that GDF15-GFRAL axis is an essential signaling node for CPT. This work reveals important physiological effects of CPT that could provide therapeutic benefits for obesity. Our strategy from virtual screening to validation, and from animal physiology to mechanistic elucidation, represents an illustrative approach potentially valuable for the next generation of translational medicine.

One may question the translational potential of CPT as a treatment for obesity due to its widely documented adverse effects in patients with adenocarcinoma of gastrointestinal origin [25]. This is less likely to happen because (1) we directly showed that the recommended anti-obesity dose of CPT (1 mg kg^−1^ day^−1^) did not provoke the documented adverse effects in obese mice, as judged by normal levels of hemoglobin, counts of white blood cell and platelet, as well as absence of diarrhea and alopecia. (2) The immune system, energy metabolism, and drug response in obese people are different from those in cancer patients [42]. For example, we show that the beneficial effects of CPT on body weight control appear not mechanistically linked to dsDNA breaks, whereas it is considered as the mechanism of action for CPT’s anticancer property [24]. This is an important consideration if one worries that CPT might cause dsDNA breaks as a treatment for obesity. (3) About 1 mg kg^−1^ day^−1^ of CPT recommended for anti-obesity purposes is equivalent to approximately one-thirtieth of the lowest dose tested in cancer patients during Phase II clinical trials, thus is much lower than those tested for anticancer purposes. These represent a major asset in support of the potential advancement of this compound to the investigational new anti-obesity drug status.

The genetic leptin-deficient *ob/ob* mice were used in this study. CPT-elevated circulating GDF15, although relatively mild in *ob/ob* mice compared to that in HFD-induced DIO mice, is able to reduce food intake and body weight in the absence of leptin. Our observations are consistent with findings that overexpression of GDF15 reduced food intake and body weight in *ob/ob* and *db/db* mice [43]. Similar findings that exogenous GDF15 reversed hyperphagia and obesity were also shown in leptin receptor-deficient ZF rats [41]. These results suggest that GDF15 functions independently of leptin signaling pathway although both hormones regulate appetite. Thus, GDF15 may have potential clinical implications for the treatment of hereditary obesity, especially in the circumstance of leptin receptor deficiency, which cannot be improved by leptin administration.

It is worth noting that only obese mice respond to CPT. This obesity-dependent effect is widely shown in mice [20,44] and is also supported by our previous work on small compounds Urolithin A [45] and Neohesperidin [46]. The underlying mechanisms of these phenomena might be different and need further investigation. In regard to CPT, low levels of GDF15 might be the reason for the absence of impact on food intake and body weight in lean mice. It is well established that the effect of GDF15 on food intake is dose dependent [37]. CPT-induced GDF15 elevations were shown in obese mice (Fig 2A, S5A Fig) but not in lean mice (S6C Fig). Thus, the mechanism underlying the obesity-dependent effect of CPT is highly probably due to serum levels of GDF15, which is relatively low and not sufficient to suppress food intake and body weight in lean mice.

We provide a mechanistic basis underlying the metabolic benefits of CPT. Using GDF15 neutralization or its receptor GFRAL deficiency in mice, we corroborated that GDF15-GFRAL signaling is indispensable for the metabolic actions of CPT. About 1 mg kg^−1^ day^−1^ of CPT moderately increases circulating levels of GDF15 (approximately 400 pg ml^−1^) and progressively reduces food intake without causing obvious adverse effects, suggesting that endogenous induction of GDF15 to a concentration range between 250 and 600 pg ml^−1^ is able to control body weight. We, therefore, recommend the range of plasma GDF15 levels for anti-obesity purposes.

Since CPT was originally tested as an anticancer drug, and some platinum-based anticancer drugs such as cisplatin and bleomycin have been shown to induce GDF15 in subjects with testicular cancer [47], one may deduce that a large number of the anticancer drugs could induce GDF15. This assumption is refuted by studies from Breen and colleagues, showing that clinically approved anticancer drugs including gemcitabine, methotrexate, paclitaxel, pemetrexed, and even the clinically approved CPT analogy CPT-11 were unable to induce GDF15 [28]. Therefore, induction of GDF15 by anticancer drugs seems not category based but individually different.

Although multiple organs and tissues contribute to the GDF15 rise in plasma [48–50], our data showed that the liver is the main source of circulating GDF15 following CPT treatment. Hepatic GDF15 production has been related to the increased levels of circulating GDF15 following sustained caloric excess as well as a consequence of acute nutritional stress [37], which indicates that the liver plays a key role in the production of GDF15. Furthermore, elegant work from the O’Rahilly lab showed that intestine and kidney are main sources for metformin-induced GDF15 in mice [51]. Recently, it has been shown that prolonged endurance exercise increases *Gdf15* expression in liver, skeletal muscle, and cardiac muscle in mice [52]. Whether the functions of hepatic GDF15 are different from those of intestinal, renal, or muscle sources merits further investigation.

GDF15 has been repeatedly shown to cause anorexia and food aversion [31,53–55] as part of the biological role of the peptide [37]. However, the roles of GDF15 in these aspects are far from conclusive. One recent study showed that recombinant human GDF15 (pharmacological), but not similar concentrations of endurance exercise induced physiological GDF15, suppresses feeding in mice, suggesting an origin-related effect of GDF15 on appetite regulation [52]. This study also indicates that the functional difference between acutely administered exogenous GDF15 and endogenous GDF15 induced by exercise or drugs may be due to the speed of GDF15 elevation. The former often leads to a fast rise in plasma GDF15, whereas the latter is relatively slow. Here, by performing CTA test in mice and pica test in rats, we observed absence of sickness behaviors indicative of nausea or malaise upon low-dose CPT (1 mg kg^−1^) treatment. Concentration is another key factor affecting the effects of GDF15. Low levels of GDF15 (<200 pg ml^−1^) is not sufficient to suppress appetite and cause food aversion [51,52] in normal mice. High levels of GDF15 were reported in cancer patients with cachexia, manifesting great loss of adipose tissue and skeletal muscle [56,57]. So far, the relationship between GDF15 and these side effects is associative rather than causative. Pathological levels of GDF15 might contribute to these adverse effects. In line with this, Hsu and colleagues observed great loss of adipose tissue and skeletal muscle when GDF15 levels were at approximately 900 pg ml^−1^ induced by 10 mg kg^−1^ of Cisplatin in mice [41], whereas skeletal muscle mass and strength were not affected or even improved when GDF15 levels were maintained approximately 400 pg ml^−1^ with 1 mg kg^−1^ of CPT, suggestive of concentration-dependent side effects of GDF15. Of note, the effect of GDF15 on skeletal muscle has been investigated in mice with GDF15 overexpressing locally in the tibialis anterior. In this way, *Gdf15* mRNA expression is increased in the skeletal muscle, manifesting muscle wasting in normal mice [58]. However, *Gdf15* mRNA is not increased in the skeletal muscle of CPT-treated mice as shown in Fig 4A. Thus, the effect of GDF15 on skeletal muscle might be concentration dependent and/or source related.

In addition to the anti-obesity property of CPT-induced GDF15 elevation, GDF15 is also implicated in life span [59], atherosclerotic cardiovascular disease [60], inflammation and immunology [61], mitochondrial disease, cancer, rheumatoid arthritis, chronic renal, and cardiac failure [43,62]. Therefore, our findings open avenues of CPT research in these fields. In summary, CPT ameliorates obesity by acting as a GDF15 inducer, providing a convincing argument that CPT may have therapeutic benefits for obesity and its associated metabolic disorders. Further study is needed to evaluate the efficacy and safety of CPT in advanced models to increase the translational impact.

## Materials and methods

### Ethics approval

All the experimental procedures were performed under the Guide for the Care and Use of Laboratory Animals: Eighth Edition (ISBN-10: 0-309-15396-4). We have complied with all relevant ethical regulations for animal testing, and all animal studies were approved by the guidelines of the ethics committee of the Northwest A&F University (Permission ID: 20181119–005).

### CMAP

CMAP project data, gene-expression profiles of cultured human cells (MCF7, PC3, SKMEL5, HL60) treated with large collection of bioactive small molecules, measured on affy U133 array platform. CMAP resource can be used to explore connections among small molecules sharing a mechanism of action, chemicals and physiological processes, diseases, and drugs. The current version (build 02; https://xenabrowser.net) of CMAP collects more than 7,000 gene-expression profiles representing 6,100 compounds. By inputting a gene-expression profile of interest and querying it against the CMAP data, a list of ranked CMAP drugs is obtained. Data shown are reversed rank scaled between 0 to 1, 1 represents the most up-regulated gene expression, and 0 represents the most down-regulated gene expression within each sample. Volcano plot analysis was performed using the OmicShare tools, a free online platform (https://www.omicshare.com/tools) for data analysis.

### Administration of CPT

Mice were orally treated with 60 μl of vehicle (0.1% Tween 80) for 3 days before CPT or vehicle treatment. Mice received 1 mg kg^−1^ of CPT (Med Chem Express, HY-16560, Shanghai, China) or vehicle (0.1% Tween 80) daily until the end of experiments.

### Animals

Male C57BL/6J mice and *ob/ob* mice were obtained from the Beijing Huafukang Bioscience (Beijing, China). *Gfral*-knockout mice were purchased from Cyagen Biosciences (Guangzhou, China). Animals were housed in the Northwest A&F University animal facility under standard conditions with 12 h of light and 12 h dark cycle with free access to food and water. Animals were randomly divided into different groups as specified. The experimental HFD diet (60% calories from fat, 20% calories from protein, and 20% calories from carbohydrate, H10060) was purchased from Beijing Huafukang Bioscience (Beijing, China).

#### Animal protocol 1: Effect of CPT on DIO mice

Male C57BL/6J mice were fed HFD for 18 weeks when their body weight reached approximately 43 g, then the DIO mice were randomly assigned for the experiments (vehicle control group, CPT treatment group, 6 mice per group). Body weight and food intake were monitored every 3 days. At the indicated time points, mice were anesthetized in chambers saturated with isoflurane after 6 h starvation and then sacrificed by cardiac puncture. After centrifugation at 6,000 rpm at 4°C for 5 min, plasma samples were separated. Organs and tissues were carefully collected, weighed, and frozen at −80°C until subsequent analysis.

#### Animal protocol 2: GDF15 antibody neutralization in *ob*/*ob* mice

GDF15 antibody neutralization was performed as previously described [43] with minor modifications. Briefly, 9-week-old *ob/ob* mice were treated with either GDF15 antibodies (5 mg kg^−1^, Abcam, ab180929, Cambridge, UK) [63] or IgG control through subcutaneous injection 24 h before CPT oral administration every other day for a period of 27 days. Body weight and food intake were monitored every 3 days. At the last day (day 27), 1 h after CPT treatment, tail blood was collected into EDTA-coated tubes, and plasma was obtained as in **Animal Protocol 1**. Organs and tissues were carefully collected, weighed, and frozen at −80°C until subsequent analysis.

#### Animal protocol 3: AAV8-mediated Gdf15 knockdown

To knock down GDF15 expression in liver, we transduced AAV8 system carrying shRNA against GDF15 (designed and synthesized by Hanbio, Shanghai, China) into mice, scramble shRNA was used as a negative control. Target sequences are listed in S2 Table. A schematic diagram for mice administration is shown in Fig 5A. In brief, DIO mice aged 13 weeks were randomly divided into 4 groups: Vehicle + AAV8-*scramble*-shRNA, CPT + AAV8-*scramble*-shRNA, Vehicle + AAV8-*Gdf15*-shRNA, and CPT + AAV8-*Gdf15*-shRNA. AAV was diluted in saline to 1 × 10^12^ vector genomes ml^−1^, and 100 μl was injected through the tail vein for each mouse. Body weight and food intake were monitored every 3 days. On the day of 21 after AAV injection, mice started to receive either vehicle or CPT at 1 mg kg^−1^ for 33 days. Then, mice were anesthetized in chambers saturated with isoflurane after 6 h starvation and sacrificed by cardiac puncture. The plasma was obtained as in **Animal Protocol 1**.

#### Animal protocol 4: CPT and high-fat diet, Gfral^−/−^ mice

Experimental cohorts of male *Gfral*^−/−^ and *Gfral*^+/+^ mice were obtained by het x het breeding pairs. A schematic diagram for mice administration is shown in Fig 7A. Male *Gfral*^+/+^ and *Gfral*^−/−^ mice, aged 8 weeks, were maintained on HFD for 5 weeks. On the day of first gavage, body weight of study groups (mean ± SEM) was 32.01 ± 1.13 g versus 32.06 ± 1.02 g for *Gfral*^+/+^ vehicle and CPT treatments, respectively, and 32.44 ± 1.31 g versus 32.24 ± 1.29 g for *Gfral*^−/−^ vehicle and CPT treatments, respectively. Each mouse received a daily gavage of either vehicle or CPT (1 mg kg^−1^) for 30 days, and their body weight and food intake were monitored every 3 days. On day 30, mice were euthanized by terminal anesthesia 1 h after gavage, and plasma was obtained as in **Animal protocol 1**. Tissues were fresh frozen on dry ice and kept at −80°C until the day of RNA extraction.

#### Animal protocol 5: Effect of single-dose CPT-11 on DIO mice

One cohort of male C57BL/6J mice (weighing 32 to 35 g) received single oral administration of vehicle or CPT-11 (Med Chem Express, HY-16562, Shanghai, China) (1 mg kg^−1^ day^−1^). After CPT-11 or vehicle treatment, tail blood was collected into EDTA-coated tubes at indicated time points, respectively.

#### Animal protocol 6: *Ob*/*ob* mice

Male *ob/ob* mice, aged 8 weeks, were housed individually with free access to water. After a 1-week acclimatization period, *ob/ob* mice were randomly split into 2 groups (*n =* 6/group) and raised under a standard chow diet with either vehicle or CPT (1 mg kg^−1^). Animals were weighed every 3 days and food consumption was measured every 3 days. At the end of 30-day testing, mice were anesthetized in chambers saturated with isoflurane after 6 h starvation and then sacrificed by cardiac puncture. The plasma was obtained as in **Animal protocol 1**.

#### Animal protocol 7: Lean mice

One cohort of male C57BL/6J mice (lean, weighing 22 to 23 g) orally received either vehicle or CPT (1 mg kg^−1^) for 30 days. Body weight and food intake were monitored every 3 days.

#### Animal protocol 8: Skeletal muscle function analysis

Kondziela’s inverted screen test was performed as previously reported [64,65]. Briefly, each mouse was put at the center of the wire mesh and rotated to an inverted position. The time when the mouse falls off was measured and scored as the following: 1, falling between 1 and 10 s; 2, between 11 and 25 s; 3, between 26 and 60 s; 4, between 60 and 90 s; 5, after 90 s. The forelimb grip strength test was performed in mice by using a grip strength meter (Columbus Instruments). The antifatigue experiment was performed as reported [66]. Briefly, mice were placed in a swimming pool filled with fresh water at 25 ± 1°C. A tin wire (5% of body weight) was loaded at the root of the mouse’s tail. The swimming period was regarded as the time spent by the mouse floating in the water, struggling, until exhausted (failed to rise to the surface of the water within 10 s).

#### Animal protocol 9: Effect of single-dose CPT upon GTT

Aged 8 weeks mice were fed 60% HFD for 4 weeks and then further split into vehicle or CPT (1 mg kg^−1^) treatment groups, given a single gavage dose at 08:00, and fasted for 6 h. At time of GTT, mice then received an intraperitoneal (IP) injection of glucose (2 g kg^−1^ of body weight) with serial measurement of glucose levels at time points indicated.

#### Animal protocol 10: Pair feeding

Pair feeding test was performed as described [20]. In brief, pair-fed vehicle-treated animals received the same amount of food as ingested by the corresponding CPT-treated group the day before. Body weight and food intake were measured daily.

#### Animal protocol 11: CTA test

CTA test was performed as described [37]. Briefly, male C57BL/6J obese mice (weighing 35 to 37 g) were singly housed and allowed ad libitum access to tap water and HFD. Mice received oral gavage with either vehicle or CPT at 1 mg kg^−1^. Mice were acclimated (up to 3 days) to drinking from 2 water bottles to confirm lack of side preference prior to habituation. Mice were then habituated to overnight water restriction (days 1 to 3) followed by 1 h water bottle presentation (2 bottles) and vehicle oral administration. On day 4 to begin conditioning, mice were given a novel 0.15% saccharin solution in both bottles instead of water for 1 h, followed by an oral administration of either vehicle, CPT (1 mg kg^−1^) or the positive control LiCl (0.15 M, 20 ml kg^−1^, Sigma). Access to saccharin water was allowed for an additional 30 min and was then changed back to water until the next restriction. Day 5 was used as a CPT washout period using the days 1 to 3 bottle protocol. A second conditioning period was performed on day 6 followed by a washout period on day 7. On day 8, a standard 2-bottle preference test (saccharin versus water) was used to assess CTA development to the saccharin solution (1 h presentation after overnight water restriction). The CTA test was performed 48 h after the last CPT administration, and volume measurements were for 1 h. Fluid intake volume was calculated for both saccharin and water. Food intake was measured at 1 h and 4 h following a single oral administration of CPT given immediately prior to the onset of the dark cycle.

#### Animal protocol 12: Pica test

Male rats aged 8 weeks were exposed to HFD in addition to normal diet for 5 weeks prior testing. One week before the onset of treatment, normal diet was removed and replaced by kaolin. Rats were kept on HFD for the entire experimental period. Then, the obese rats were divided into 3 body weight–matched groups (*n =* 5 per group), received either 1 mg kg^−1^ of CPT, vehicle, or Cisplatin (as a positive control based on previous studies [67,68]) for 6 consecutive days. Food, kaolin, and body weight were measured every day.

#### Animal protocol 13: GDF15 antibody validation

Three-month-old C57BL/6 mice were treated with either vehicle or recombinant GDF15 (0.1 mg kg^−1^, 8944-GD, R&D, the dose was selected based on previous reports on the efficiency of GDF15 neutralizing antibody [69,70]) (IP injection, every other day) for 12 days. On day 7, the GDF15-treated mice were divided into 2 groups (*n* = 5 per group), receiving either GDF15 antibody (5 mg kg^−1^) or IgG control (IP injection, every other day).

#### Animal protocol 14: Safety evaluation of CPT in DIO mice

Male C57BL/6J mice weighing 36 to 38 g were sorted into different treatment groups (vehicle versus CPT), with each group having comparable starting body weight.

### Dose conversions

The calculation of CPT dose between mice and humans is based on the FDA guidance [27]. Briefly, we derived the following equation for converting mouse dose (mg kg^−1^) to the HED based on body weights and the allometric exponent (b):

$$HED\;(mg/m^{2})=[animal\;NOAEL\times {\left(W_{animal}/W_{human}\right)}^{\left(1‐b\right)}]\times Km$$

where b is allometric exponent, W is body weight in kilogram (kg), and Km is factor for converting mg kg^−1^ dose to mg/m^2^ dose. According to the guideline, HED calculations is based on b = 0.67, Km = 37. Therefore, the HED of 1 mg kg^−1^ CPT in a 30-g mouse is equivalent to 3.01 mg/m^2^ for a 60-kg person.

### Pharmacokinetics of CPT

Pharmacokinetics of CPT was performed as described [71] with minor modifications. One cohort of male C57BL/6J mice (weighing 33 to 35 g) orally received CPT (1 mg kg^−1^). Plasma samples were collected at indicated points (0, 1, 4, 6, and 12 h) after oral administration and stored at −20°C for further processing. CPT concentrations in the mice plasma were quantified by a Shimadzu high-performance liquid chromatography (HPLC) system.

### Gamma H2AX measurement

γH2AX levels in AML12 cells, Hepa1-6 cells, and mouse livers were determined by ELISA-based pharmacodynamic assay (4418-096-K, R&D) according to the manufacturer’s instructions.

### Calorimetry

This was determined by using Oxymax/CLAMS as described [72]. Briley, mice were placed in individual metabolic cages and allowed to acclimate for a period of 48 h before data collection every 30 min. EE was measured with an Oxymax/Comprehensive Lab Animal Monitoring System (Columbus Instruments, Columbus, OH). Measurements were performed for the dark (from 18:00 to 06:00) or light (from 06:00 to 18:00) period under ad libitum feeding conditions.

### Body temperature

The body temperature was recorded by a rectal probe (BAT-12, Physitemp, Clifton, NJ, USA).

### Thermal imaging experiments

Thermal imaging experiments were carried out in a closed room at a constant temperature of 22°C with tolerance of 1°C. An infrared (IR) camera (FLIR C2, Estonia) was used to examine the mouse body temperature.

### Plasma analysis

The plasma biochemical parameters include ALT, AST, TG, TC, CREA, and BUN were measured using an automatic biochemical analyzer (Hitachi 7180, Tokyo, Japan) in the Yangling Demonstration Zone Hospital. Levels of mouse plasma GDF15 and medium GDF15 released from cultured hepatocytes were measured using a mouse GDF15 enzyme-linked immunosorbent assay (MGD150, R&D, Minneapolis, USA), according to the manufacturer’s instructions. The TG of liver tissues was measured using a TG quantification kit (Jiancheng, Nanjing, Jiangsu, China) followed by the manufacturer’s instructions.

### GTT and ITT

For GTT, overnight-fasted mice received an IP injection of glucose (2 g kg^−1^ of body weight). For ITT, 6 h–fasted mice received an IP injection of insulin (Humulin R, Eli Lilly, Indianapolis, USA) (1 units kg^−1^ of body weight). Tail blood glucose levels were measured at indicated points (0, 30, 60, 90, and 120 min) after injection.

### Cell culture

AML12 cell lines were obtained from American Type Culture Collection (ATCC). Hepatocytes were plated onto 12-well plates to 85% confluency and allowed to adhere overnight in DMEM: F12 (SH30022.01, HyClone, CT, USA) supplemented to a final concentration of 10% fetal calf bovine plasma (FBS) (Z7186FBS-500, ZETA LIFE, CA, USA), 1% antibiotic–antimycotic solution. CPT was added for 24 h at the concentrations indicated in the figure legends. siRNA experiments were performed as described [73]. Briefly, AML12 cell lines were plated onto 12-well plates. Approximately 10 h later, 10 pmol of *Atf4* siRNA, or *Chop* siRNA, or *scramble* Negative Control siRNA (GenePharma, Shanghai, China) was added with Lipofectamine RNAiMAX Transfection Reagent (GenePharma, Shanghai, China) and Opti-MEM I reduced plasma medium (31985070, Gibco) into the medium according to the manufacturer’s instructions. The next morning, medium was replaced, and CPT (1 μM) was added. Approximately 24 h after CPT addition, the medium was collected and stored at −80°C for further analysis.

### Real-time qPCR and western blots

Total RNA extraction from tissues, reverse transcription, and qPCR were performed as previously described [45]. Primer pairs for qPCR were listed in S3 Table. Western blots were performed as described [74].

### In vivo c-Fos and GFRAL detection and quantification upon CPT treatment

Immunofluorescence processing of brain sections was performed as previously reported [41] with minor modifications. DIO mice (weighing 40 to 42 g) received oral gavage with either vehicle or CPT at 1 mg kg^−1^. After 1 h, animals were anesthetized and perfused intracardially with 4% paraformaldehyde. Whole brains were isolated, fixed at 4°C overnight, and cryoprotected in 30% sucrose before sectioning. The number of c-Fos positive cells in each brain region was quantified manually in 1 to 3 sections from each animal per condition. Double immunofluorescence staining of c-Fos (sc-52, Santa Cruz, Texas, USA) and GFRAL (AF5728, R&D, Minneapolis, USA) were performed according to the manufacturer’s instructions. Double immunofluorescence staining demonstrated a direct interaction between c-Fos and GFRAL. The images were acquired using Bio TEX Software.

### Statistical analysis

Values are presented as mean ± SEM. Statistical comparisons for 2 groups were performed by the Student *t* test and for more than 2 groups, by one-way ANOVA followed by Fisher’s LSD post hoc test with SPSS software (v.20.0; IBM, Armonk NY, USA), * *P* < 0.05; ** *P* < 0.01; *** *P* < 0.001.

## Supporting information

S1 Fig Distribution of enrichment scores used as CMAP queries. Distribution of enrichment scores of individual small molecules obtained from CMAP database in **(A)** MCF7 and **(B)** PC3 cells. In this study, we chose a list of small molecules with an enrichment score over 0.6. The underlying data for this figure can be found in **S1 Data**. CMAP, Connectivity Map; CPT, Camptothecin.
(TIF)

S2 Fig Concentration-time profiles of CPT in plasma. Plasma levels of CPT in DIO mice were measured 1, 4, 6, or 12 h after a single oral dose of 1 mg kg^−1^ of CPT. Data are presented as mean ± SEM. *n =* 6 per group. The underlying data for this figure can be found in **S1 Data**. CPT, Camptothecin; DIO, diet-induced obese.
(TIF)

S3 Fig Effect of CPT on plasma GDF15 levels in *ob/ob* mice. Plasma levels of GDF15 in *ob/ob* mice were measured 1, 2, 6, 12, or 24 h after a single oral dose of 1 mg kg^−1^ of CPT. Data are presented as mean ± SEM. *n =* 5 per group. The underlying data for this figure can be found in **S1 Data**. CPT, Camptothecin; GDF15, growth differentiation factor 15.
(TIF)

S4 Fig Effect of single-dose CPT-11 on plasma GDF15 levels in DIO mice. DIO mice were subjected to single oral administration of vehicle or CPT-11 (1 mg kg^−1^ day^−1^). Plasma levels of GDF15 were measured at time points indicated. Data are presented as mean ± SEM. *n =* 5 per group. The underlying data for this figure can be found in **S1 Data**. CPT, Camptothecin; DIO, diet-induced obese; GDF15, growth differentiation factor 15.
(TIF)

S5 Fig CPT ameliorates obesity in *ob/ob* mice. **(A-C) Animal protocol 6**: *Ob/ob* mice were subjected to oral administration of vehicle or CPT (1 mg kg^−1^ day^−1^) for 30 days. **(A)** Plasma levels of GDF15 at indicated time points. **(B)** Cumulative food intake. **(C)** Body weight and representative pictures of *ob/ob* mice treated with CPT or vehicle. Data are presented as mean ± SEM. *n =* 6 per group. The underlying data for this figure can be found in **S1 Data**. CPT, Camptothecin; GDF15, growth differentiation factor 15.
(TIF)

S6 Fig Effects of CPT on body weight and food intake in lean mice. **(A-C) Animal protocol 7**: lean mice (body weight approximately 22 g) received vehicle or CPT (1 mg kg^−1^ day^−1^) for 30 days. **(A)** Cumulative food intake. **(B)** Consecutive body weight and representative mouse pictures at the end of treatment. **(C)** Plasma levels of GDF15 after 30-day treatment. Data are presented as mean ± SEM. *n =* 6 per group. The underlying data for this figure can be found in **S1 Data**. CPT, Camptothecin; GDF15, growth differentiation factor 15.
(TIF)

S7 Fig Effects of CPT on tissue weight and plasma parameters in *ob/ob* mice. **(A-H) Animal protocol 6**: **(A)** Tissue weights of iWAT, eWAT, and BAT in CPT-treated mice and controls. **(B)** Plasma levels of TG and TC. **(C)** Liver weights. **(D)** Hepatic TG and TC contents. **(E)** Plasma levels of ALT and AST. **(F)** Weights of kidney and spleen. **(G-H)** Tissue weights of GAS, QUA, SOL, and EDL. Data are presented as mean ± SEM. *n =* 6 per group. The underlying data for this figure can be found in **S1 Data**. ALT, alanine aminotransferase; AST, aspartate aminotransferase; BAT, brown adipose tissue; CPT, Camptothecin; EDL, extensor digitorum longus; eWAT, epididymal white adipose tissue; GAS, gastrocnemius; iWAT, inguinal white adipose tissue; QUA, quadriceps; SOL, soleus; TC, total cholesterol; TG, triglyceride.
(TIF)

S8 Fig Effects of CPT on tissue weight in DIO mice. **(A-C) Animal protocol 1**: **(A)** Weights of kidney and spleen. **(B-C)** Tissue weights of GAS, QUA, SOL, and EDL. Data are presented as mean ± SEM. *n =* 6 per group. The underlying data for this figure can be found in **S1 Data**. CPT, Camptothecin; DIO, diet-induced obese; EDL, extensor digitorum longus; GAS, gastrocnemius; QUA, quadriceps; SOL, soleus.
(TIF)

S9 Fig CPT treatment improves exercise capacity in DIO mice. **(A-C) Animal protocol 8**: **(A)** Kondziela’s inverted screen test after 17-day CPT treatment. **(B)** Grip strength test. Forelimb (2 paws) grip force measurements after 21-day CPT treatment. **(C)** Effect of CPT on the weight-bearing swimming time in mice. Data are presented as mean ± SEM. *n* = 6 per group. The underlying data for this figure can be found in **S1 Data**. CPT, Camptothecin; DIO, diet-induced obese.
(TIF)

S10 Fig CPT improves glucose tolerance in *ob/ob* mice. **(A-B) Animal protocol 6**: (A) GTT was performed after 16-day treatment and AUC of GTT. **(B)** ITT was performed after 23-day treatment and AUC of ITT. Data are presented as mean ± SEM. *n =* 6 per group. The underlying data for this figure can be found in **S1 Data**. AUC, area under the curve; CPT, Camptothecin; GTT, glucose tolerance test; ITT, insulin tolerance test.
(TIF)

S11 Fig Effects of single-dose CPT on glucose tolerance in DIO mice. **Animal protocol 9**: GTT was performed in mice given single dose of oral CPT (1 mg kg^−1^) and AUC of GTT. The underlying data for this figure can be found in **S1 Data**. AUC, area under the curve; CPT, Camptothecin; DIO, diet-induced obese; GTT, glucose tolerance test.
(TIF)

S12 Fig Effects of CPT on plasma BUN and CREA in DIO mice and *ob/ob* mice. **(A-B) Animal protocol 1**: **(A-B)** Plasma levels of BUN and CREA in DIO mice. **(C-D) Animal protocol 5**: **(C-D)** Plasma levels of BUN and CREA in *ob/ob* mice. Data are presented as mean ± SEM. *n =* 6 per group. The underlying data for this figure can be found in **S1 Data**. BUN, blood urea nitrogen; CPT, Camptothecin; CREA, creatinine; DIO, diet-induced obese.
(TIF)

S13 Fig Effects of CPT on body weight changes under pair-feeding regime. **(A-C) Animal protocol 10**: **(A)** The average of 9-day food intake (g day^−1^). The average daily food intake of the pair-fed vehicle groups was the same as for CPT-treated mice, an extra vehicle group were allowed ad libitum access to food. **(B-C)** Consecutive body weight and changes of body weight. Data are presented as mean ± SEM. *n =* 6 per group. The underlying data for this figure can be found in **S1 Data**. CPT, Camptothecin.
(TIF)

S14 Fig CPT treatment does not alter EE in DIO mice. The EE of CPT-treated and vehicle control mice was shown in **(A)** uncorrected, **(B)** corrected for body mass, and **(C)** corrected for lean mass. **(D)** The body temperature of mice placed at room temperature (21°C) after 9 days of treatment. **(E)** Representative infrared images of mice. Images are captured using the rainbow high-contrast color palette in FLIR Research. *n =* 6 per group. The underlying data for this figure can be found in **S1 Data**. CPT, Camptothecin; DIO, diet-induced obese; EE, energy expenditure.
(TIF)

S15 Fig CTA test. **(A-C) Animal protocol 11**: Cumulative food intake measured between 1 and 4 h post-CPT oral treatment as total grams **(A)** or percent (%) of vehicle control **(B)**. **(C)** Saccharin and water consumption. Obese mice received either vehicle, CPT, or LiCl (positive control). Data are presented as mean ± SEM and analyzed using a two-way ANOVA with Bonferroni multiple comparison post-test to compare proportion of saccharin water and water consumption between groups of CPT or LiCl treatment to vehicle. *** (saccharin) or ^###^ (water) *P* < 0.001. *n =* 6 per group. The underlying data for this figure can be found in **S1 Data**. CPT, Camptothecin; CTA, conditioned taste aversion.
(TIF)

S16 Fig Effects of CPT and Cisplatin on pica, food intake, and body weight in rats. **(A-E) Animal protocol 12**: Rats were treated with vehicle, CPT (1 mg kg^−1^, gavage), or Cisplatin (6 mg kg^−1^, IP injection) for 6 days. **(A)** Kaolin intake and **(B)** the mean daily kaolin intake; **(C)** food intake and **(D)** the mean daily food consumption. **(E)** Cumulative change in body weight from baseline (g). Data are presented as mean ± SEM. * *P* < 0.05, ** *P* < 0.01, *** *P* < 0.001. *n* = 5 per group. The underlying data for this figure can be found in **S1 Data**. CPT, Camptothecin; IP, intraperitoneal.
(TIF)

S17 Fig GDF15 antibody validation. **(A-B) Animal protocol 13**: Three-month-old C57BL/6 mice were treated with either vehicle or GDF15 (0.1 mg kg^−1^) (IP injection, every other day) for 12 days. On day 7, the GDF15-treated mice were divided into 2 groups (*n* = 5 per group), receiving either GDF15 antibody (5 mg kg^−1^) or IgG control (IP injection, every other day). **(A)** Percentage change in body weight from baseline (%). **(B)** Cumulative food intake. Data are presented as mean ± SEM. The underlying data for this figure can be found in **S1 Data**. GDF15, growth differentiation factor 15; IgG, immunoglobulin G; IP, intraperitoneal.
(TIF)

S18 Fig ISR detection in ileum of CPT-treated DIO mice. Immunoblot analysis of ATF4 and CHOP relative to β-actin in ileum of DIO mice treated with 1 mg kg^−1^ of CPT for 30 days (*n* = 3). The original blot for this figure can be found in **S2 Raw Image**. ATF4, activating transcription factor 4; CHOP, C/EBP homologous protein; CPT, Camptothecin; DIO, diet-induced obese; ISR, integrated stress response.
(TIF)

S19 Fig Effects of CPT on dsDNA breaks in mouse livers and hepatocytes. Levels of γH2AX in **(A)** liver of DIO mice treated with 1 mg kg^−1^ of CPT for 30 days, and **(B)** AML 12 cells and Hepa1-6 cells treated with 1 μM CPT for 24 h. Data are presented as mean ± SEM. *n* = 6 per group. The underlying data for this figure can be found in **S1 Data**. CPT, Camptothecin; DIO, diet-induced obese.
(TIF)

S1 Raw Image Original blot contains Fig 4F–4H and 4K. (TIF)

S2 Raw Image Original blot contains S18 Fig. (TIF)

S1 Table Safety evaluation of CPT in DIO mice received either single acute or 30-day chronic treatment. (DOCX)

S2 Table List of shRNA oligonucleotides for gene knockdown. (DOCX)

S3 Table Primers used for gene amplification. (DOCX)

S1 Data Contains underlying data for Figs 1B–1D; 2A–2J; 3B–3J; 4A–4E, 4I, 4J, 4L, 4M; 5B–5K; 6B; 7B–7H; S1A, S1B; S2; S3; S4; S5A–S5C; S6A–S6C; S7A–S7H; S8A–S8C; S9A–S9C; S10A, S10B; S11; S12A–S12D; S13A–S13C; S14A–S14D; S15A–S15C; S16A–S16E; S17A, S17B; S19A, S19B. (XLSX)

## Abbreviations

ALT: alanine aminotransferase
AP: area postrema
AST: aspartate aminotransferase
ATF4: activating transcription factor 4
BAT: brown adipose tissue
BUN: blood urea nitrogen
CHOP: C/EBP homologous protein
CMAP: Connectivity Map
CPT: Camptothecin
CREA: creatinine
CTA: conditioned taste aversion
DIO: diet-induced obese
EDL: extensor digitorum longus
EE: energy expenditure
eWAT: epididymal white adipose tissue
GAS: gastrocnemius
GDF15: growth differentiation factor 15
GDNF: glial-derived neurotrophic factor
GFRAL: GDNF receptor alpha-like
GTT: glucose tolerance test
HED: human equivalent dose
HFD: high-fat diet
IgG: immunoglobulin G
ISR: integrated stress response
ITT: insulin tolerance test
iWAT: inguinal white adipose tissue
NTS: nucleus tractus solitarius
QUA: quadriceps
SOL: soleus
TC: total cholesterol
TG: triglyceride
WT: wild-type
