# Supplementary figures and images for: Camptothecin effectively treats obesity in mice through GDF15 induction
Source: PLoS Biol. 2022 Feb 24;20(2):e3001517. doi: 10.1371/journal.pbio.3001517 (PMC8870521; doi:10.1371/journal.pbio.3001517)

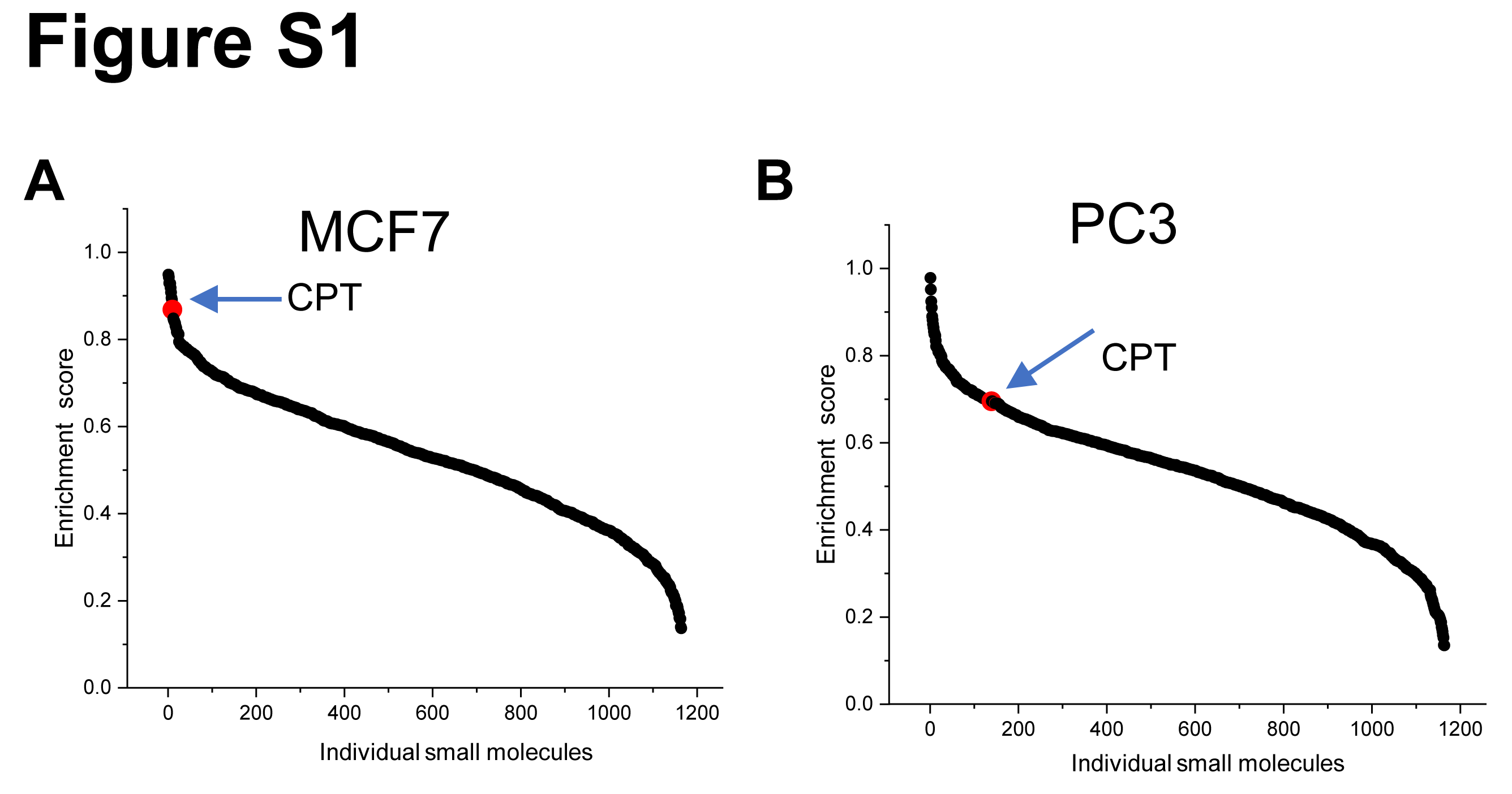

Supplement: S1 Fig — Distribution of enrichment scores of individual small molecules obtained from CMAP database in (A) MCF7 and (B) PC3 cells. In this study, we chose a list of small molecules with an enrichment score over 0.6. The underlying data for this figure can be found in S1 Data. CMAP, Connectivity Map; CPT, Camptothecin. (TIF) [file pbio.3001517.s001.tif]

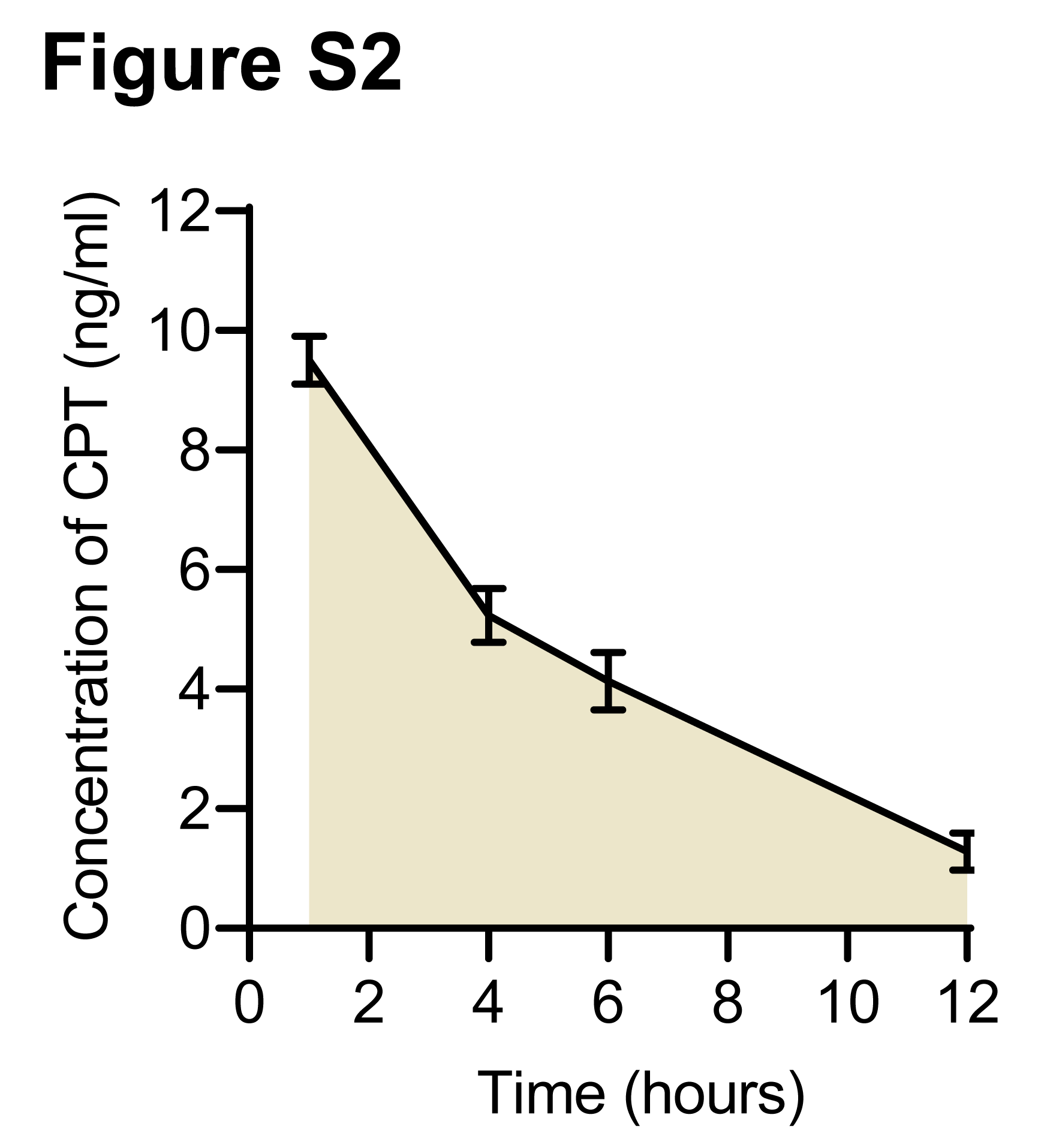

Supplement: S2 Fig — Plasma levels of CPT in DIO mice were measured 1, 4, 6, or 12 h after a single oral dose of 1 mg kg−1 of CPT. Data are presented as mean ± SEM. n = 6 per group. The underlying data for this figure can be found in S1 Data. CPT, Camptothecin; DIO, diet-induced obese. (TIF) [file pbio.3001517.s002.tif]

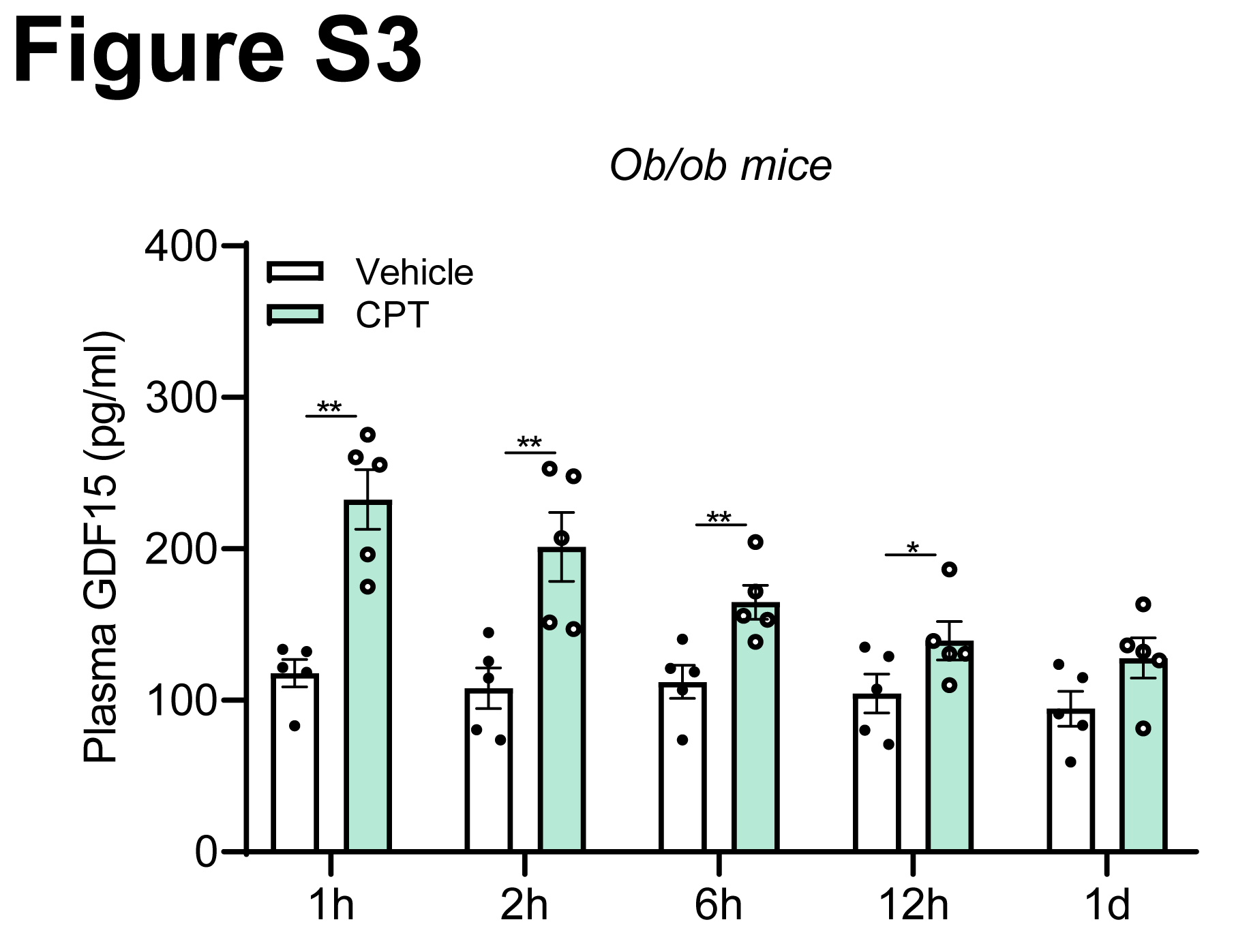

Supplement: S3 Fig — Plasma levels of GDF15 in ob/ob mice were measured 1, 2, 6, 12, or 24 h after a single oral dose of 1 mg kg−1 of CPT. Data are presented as mean ± SEM. n = 5 per group. The underlying data for this figure can be found in S1 Data. CPT, Camptothecin; GDF15, growth differentiation factor 15. (TIF) [file pbio.3001517.s003.tif]

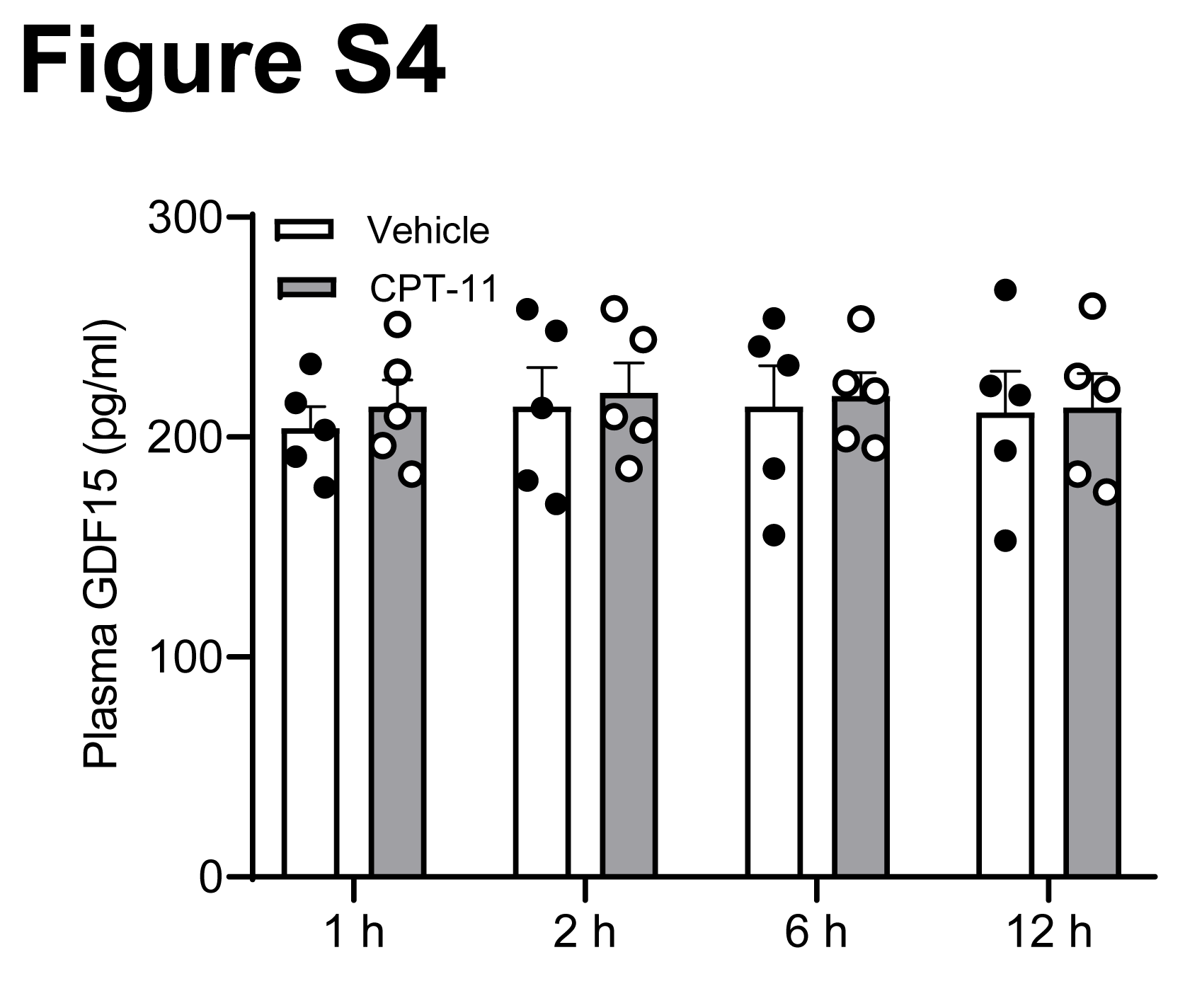

Supplement: S4 Fig — DIO mice were subjected to single oral administration of vehicle or CPT-11 (1 mg kg−1 day−1). Plasma levels of GDF15 were measured at time points indicated. Data are presented as mean ± SEM. n = 5 per group. The underlying data for this figure can be found in S1 Data. CPT, Camptothecin; DIO, diet-induced obese; GDF15, growth differentiation factor 15. (TIF) [file pbio.3001517.s004.tif]

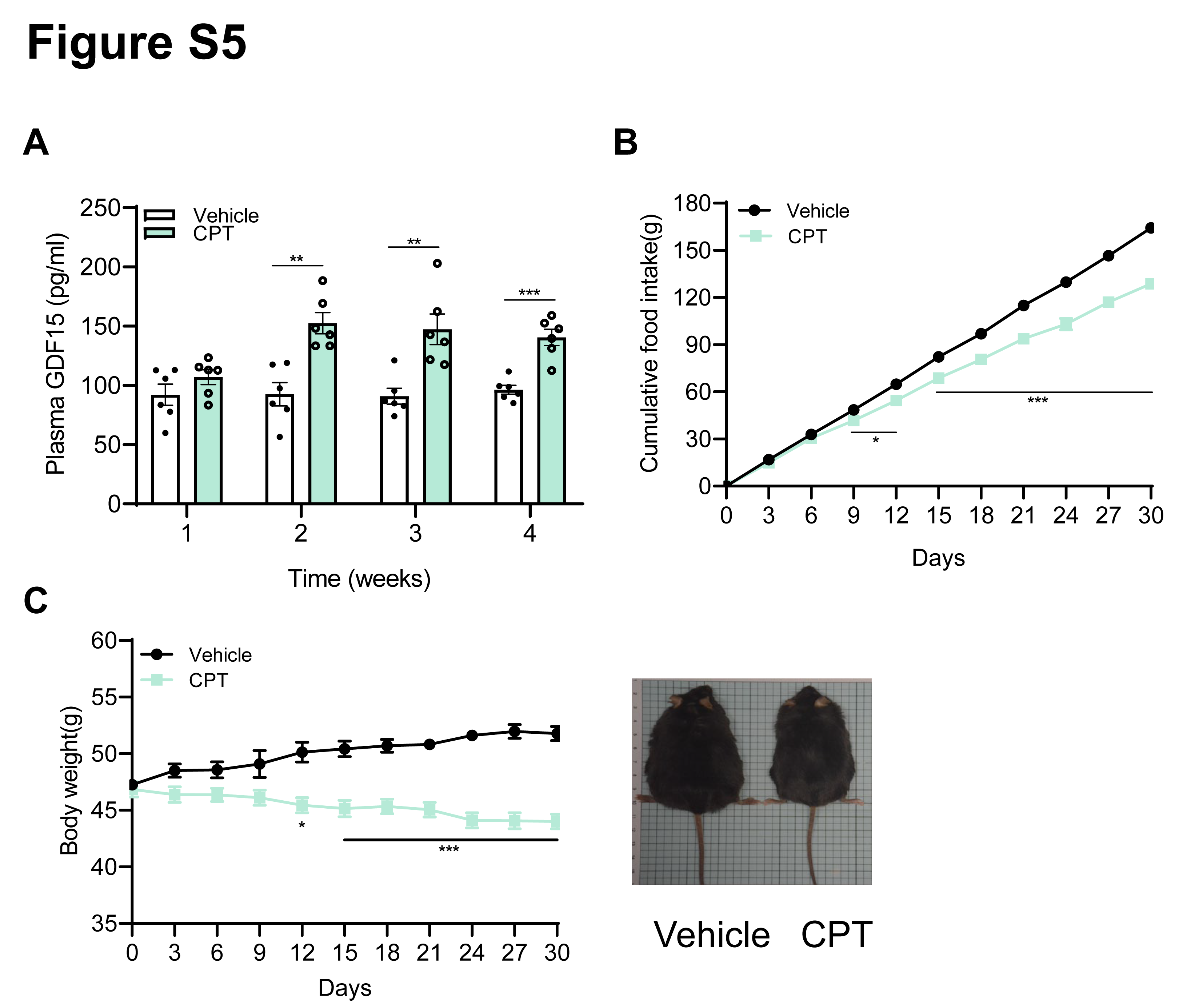

Supplement: S5 Fig — (A-C) Animal protocol 6: Ob/ob mice were subjected to oral administration of vehicle or CPT (1 mg kg−1 day−1) for 30 days. (A) Plasma levels of GDF15 at indicated time points. (B) Cumulative food intake. (C) Body weight and representative pictures of ob/ob mice treated with CPT or vehicle. Data are presented as mean ± SEM. n = 6 per group. The underlying data for this figure can be found in S1 Data. CPT, Camptothecin; GDF15, growth differentiation factor 15. (TIF) [file pbio.3001517.s005.tif]

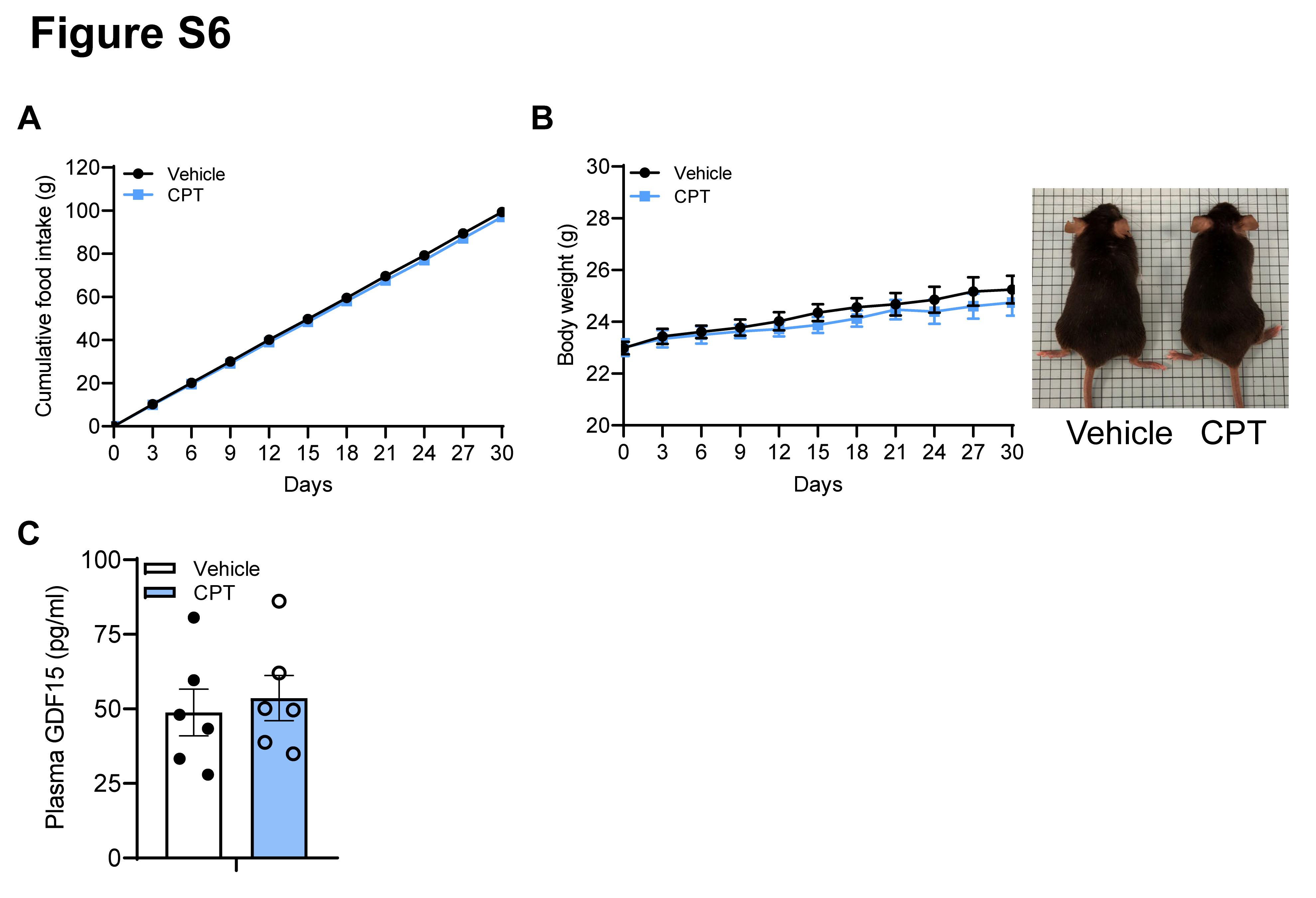

Supplement: S6 Fig — (A-C) Animal protocol 7: lean mice (body weight approximately 22 g) received vehicle or CPT (1 mg kg−1 day−1) for 30 days. (A) Cumulative food intake. (B) Consecutive body weight and representative mouse pictures at the end of treatment. (C) Plasma levels of GDF15 after 30-day treatment. Data are presented as mean ± SEM. n = 6 per group. The underlying data for this figure can be found in S1 Data. CPT, Camptothecin; GDF15, growth differentiation factor 15. (TIF) [file pbio.3001517.s006.tif]

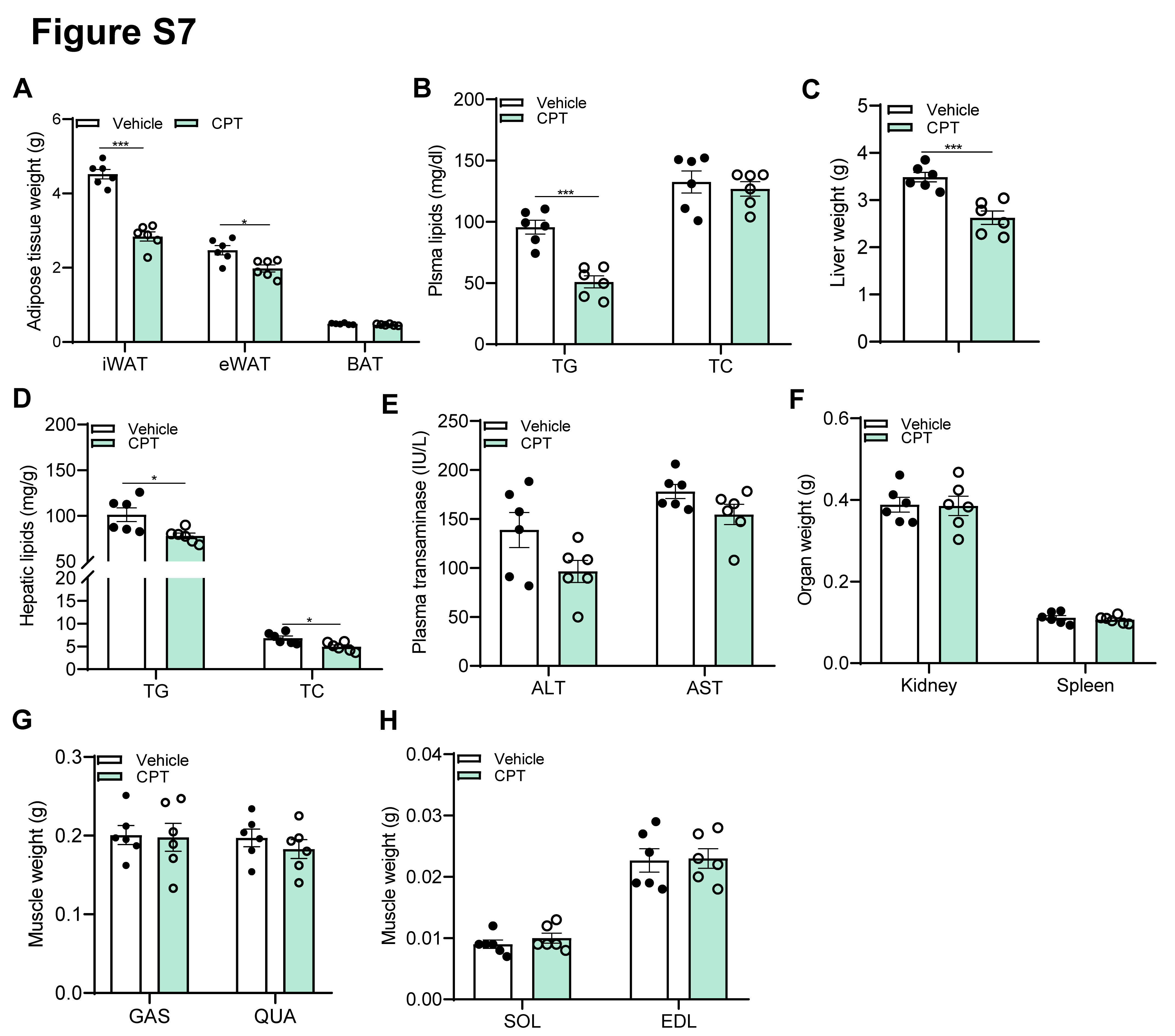

Supplement: S7 Fig — (A-H) Animal protocol 6: (A) Tissue weights of iWAT, eWAT, and BAT in CPT-treated mice and controls. (B) Plasma levels of TG and TC. (C) Liver weights. (D) Hepatic TG and TC contents. (E) Plasma levels of ALT and AST. (F) Weights of kidney and spleen. (G-H) Tissue weights of GAS, QUA, SOL, and EDL. Data are presented as mean ± SEM. n = 6 per group. The underlying data for this figure can be found in S1 Data. ALT, alanine aminotransferase; AST, aspartate aminotransferase; BAT, brown adipose tissue; CPT, Camptothecin; EDL, extensor digitorum longus; eWAT, epididymal white adipose tissue; GAS, gastrocnemius; iWAT, inguinal white adipose tissue; QUA, quadriceps; SOL, soleus; TC, total cholesterol; TG, triglyceride. (TIF) [file pbio.3001517.s007.tif]

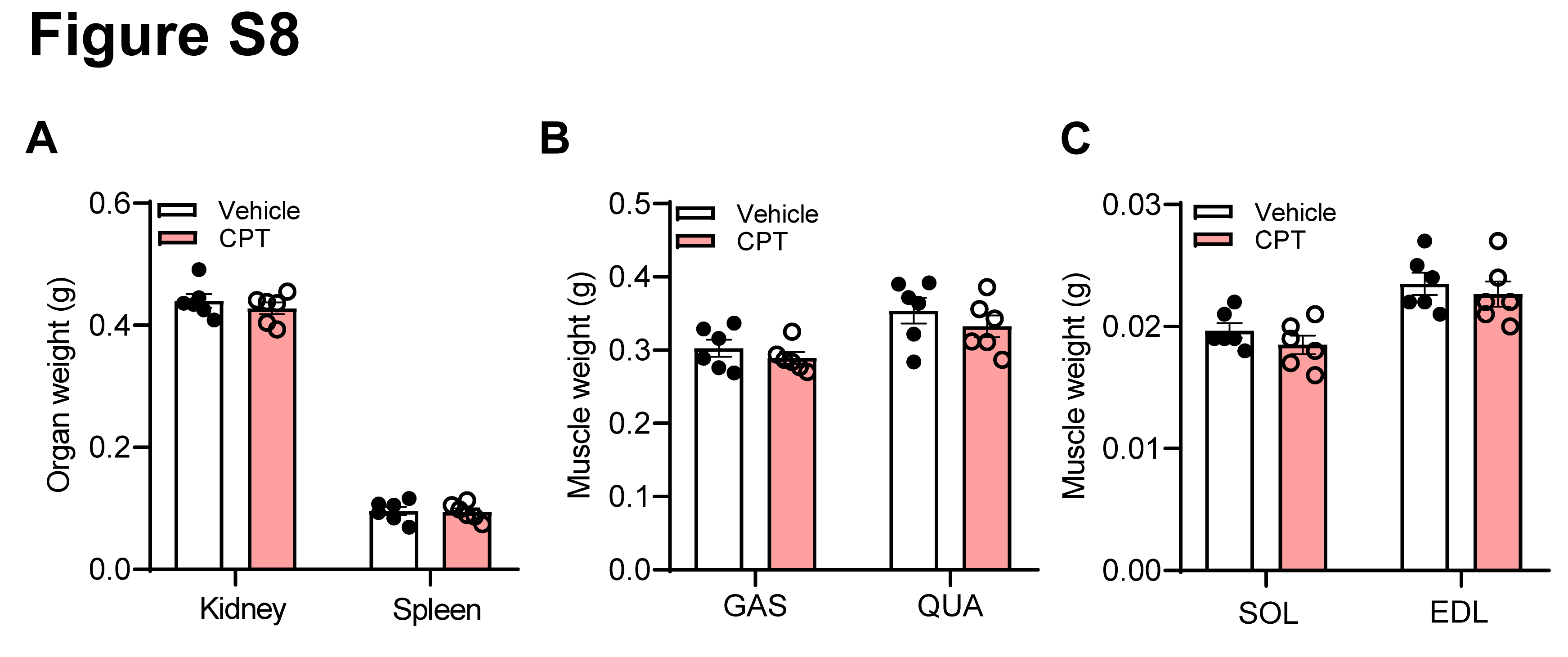

Supplement: S8 Fig — (A-C) Animal protocol 1: (A) Weights of kidney and spleen. (B-C) Tissue weights of GAS, QUA, SOL, and EDL. Data are presented as mean ± SEM. n = 6 per group. The underlying data for this figure can be found in S1 Data. CPT, Camptothecin; DIO, diet-induced obese; EDL, extensor digitorum longus; GAS, gastrocnemius; QUA, quadriceps; SOL, soleus. (TIF) [file pbio.3001517.s008.tif]

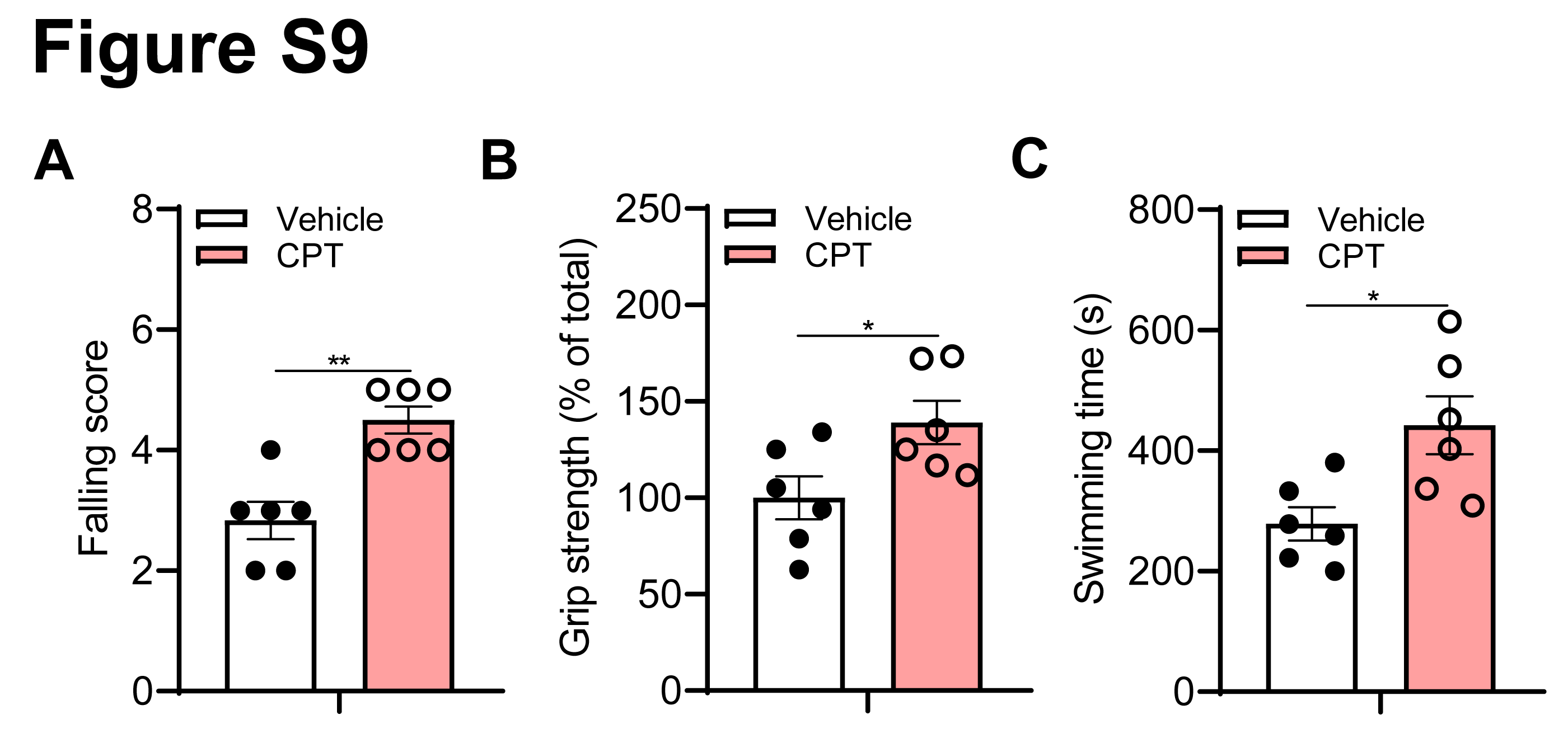

Supplement: S9 Fig — (A-C) Animal protocol 8: (A) Kondziela’s inverted screen test after 17-day CPT treatment. (B) Grip strength test. Forelimb (2 paws) grip force measurements after 21-day CPT treatment. (C) Effect of CPT on the weight-bearing swimming time in mice. Data are presented as mean ± SEM. n = 6 per group. The underlying data for this figure can be found in S1 Data. CPT, Camptothecin; DIO, diet-induced obese. (TIF) [file pbio.3001517.s009.tif]

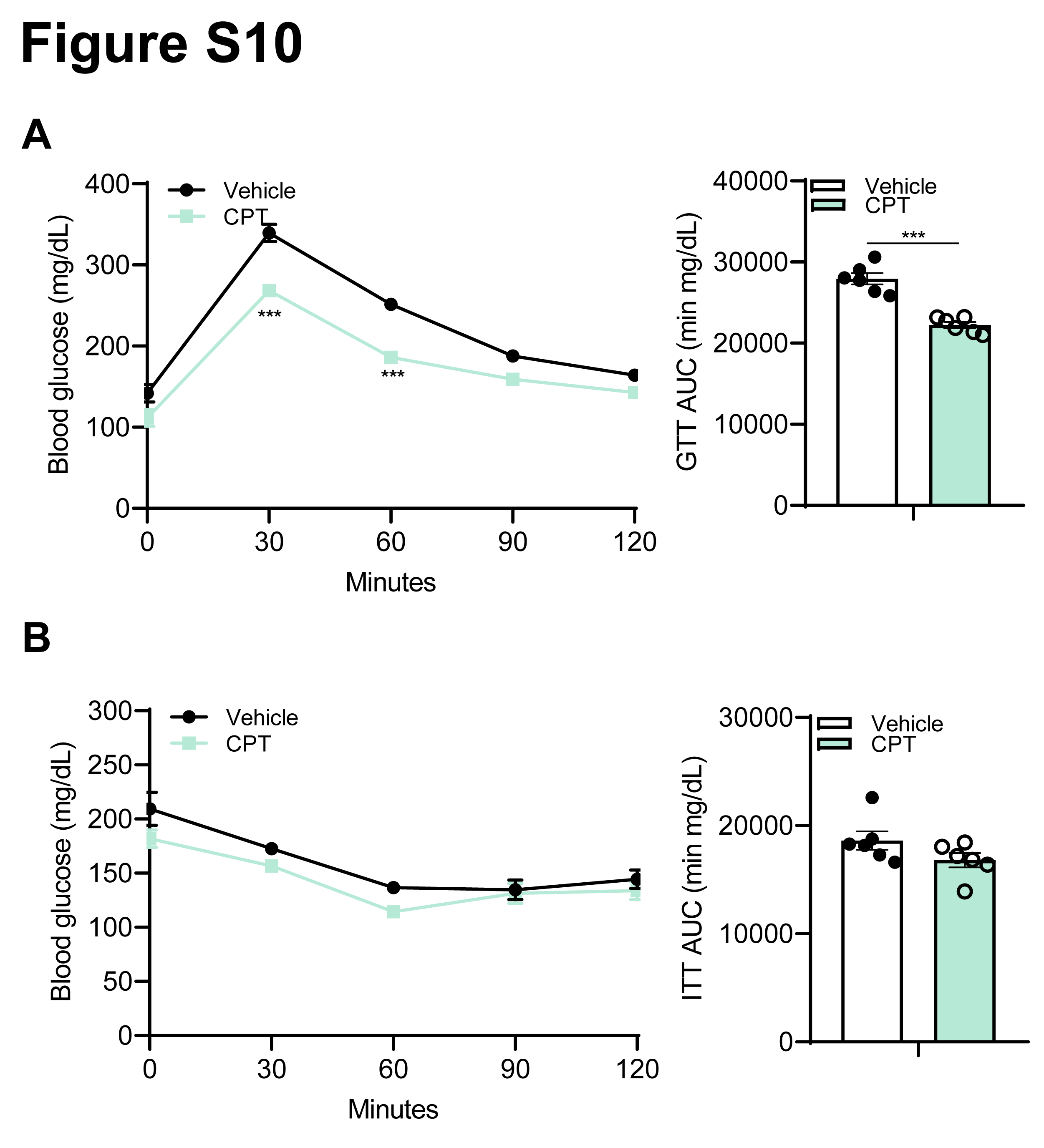

Supplement: S10 Fig — (A-B) Animal protocol 6: (A) GTT was performed after 16-day treatment and AUC of GTT. (B) ITT was performed after 23-day treatment and AUC of ITT. Data are presented as mean ± SEM. n = 6 per group. The underlying data for this figure can be found in S1 Data. AUC, area under the curve; CPT, Camptothecin; GTT, glucose tolerance test; ITT, insulin tolerance test. (TIF) [file pbio.3001517.s010.tif]

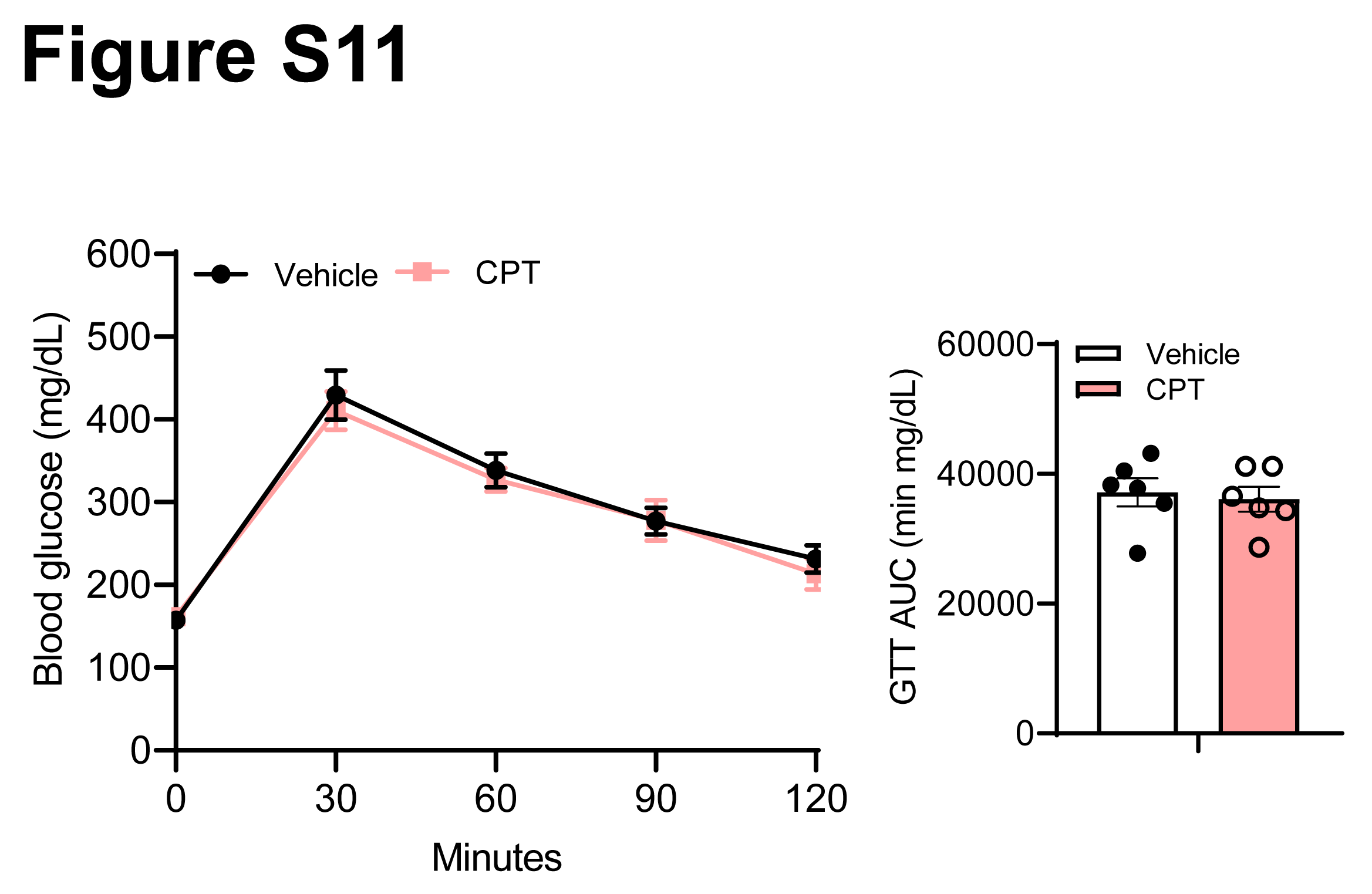

Supplement: S11 Fig — Animal protocol 9: GTT was performed in mice given single dose of oral CPT (1 mg kg−1) and AUC of GTT. The underlying data for this figure can be found in S1 Data. AUC, area under the curve; CPT, Camptothecin; DIO, diet-induced obese; GTT, glucose tolerance test. (TIF) [file pbio.3001517.s011.tif]

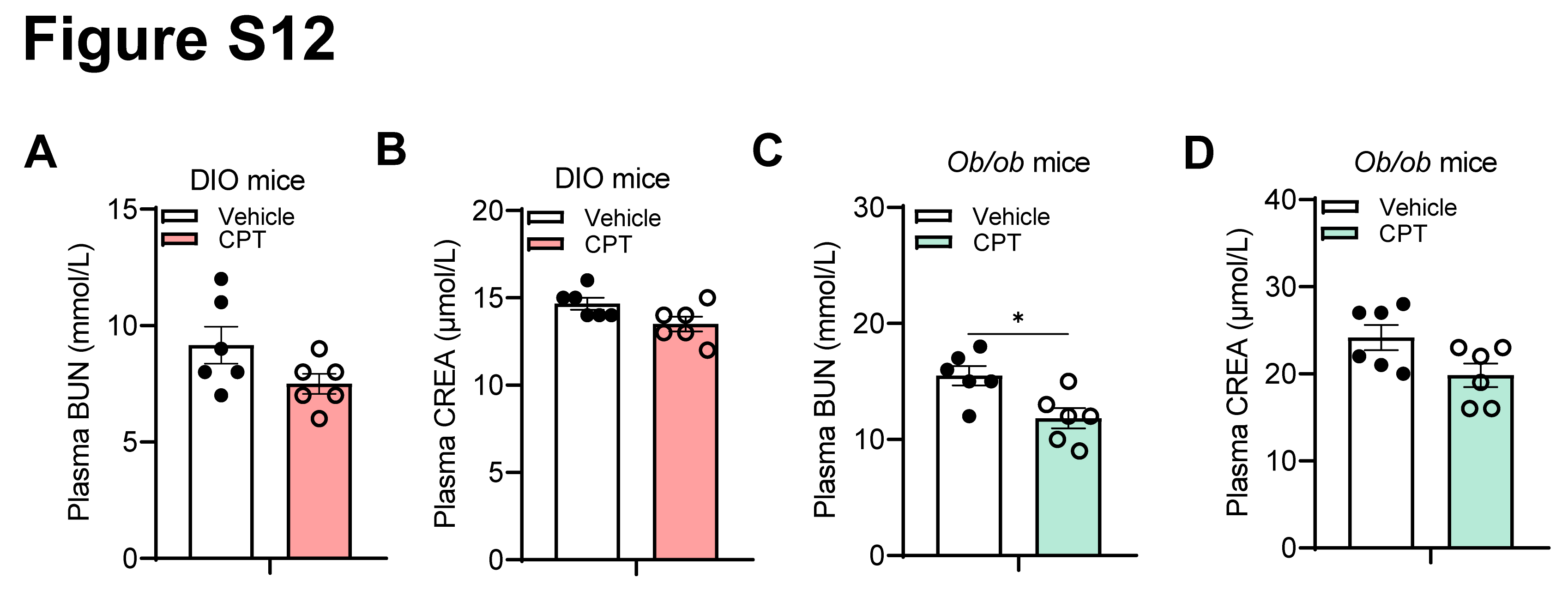

Supplement: S12 Fig — (A-B) Animal protocol 1: (A-B) Plasma levels of BUN and CREA in DIO mice. (C-D) Animal protocol 5: (C-D) Plasma levels of BUN and CREA in ob/ob mice. Data are presented as mean ± SEM. n = 6 per group. The underlying data for this figure can be found in S1 Data. BUN, blood urea nitrogen; CPT, Camptothecin; CREA, creatinine; DIO, diet-induced obese. (TIF) [file pbio.3001517.s012.tif]

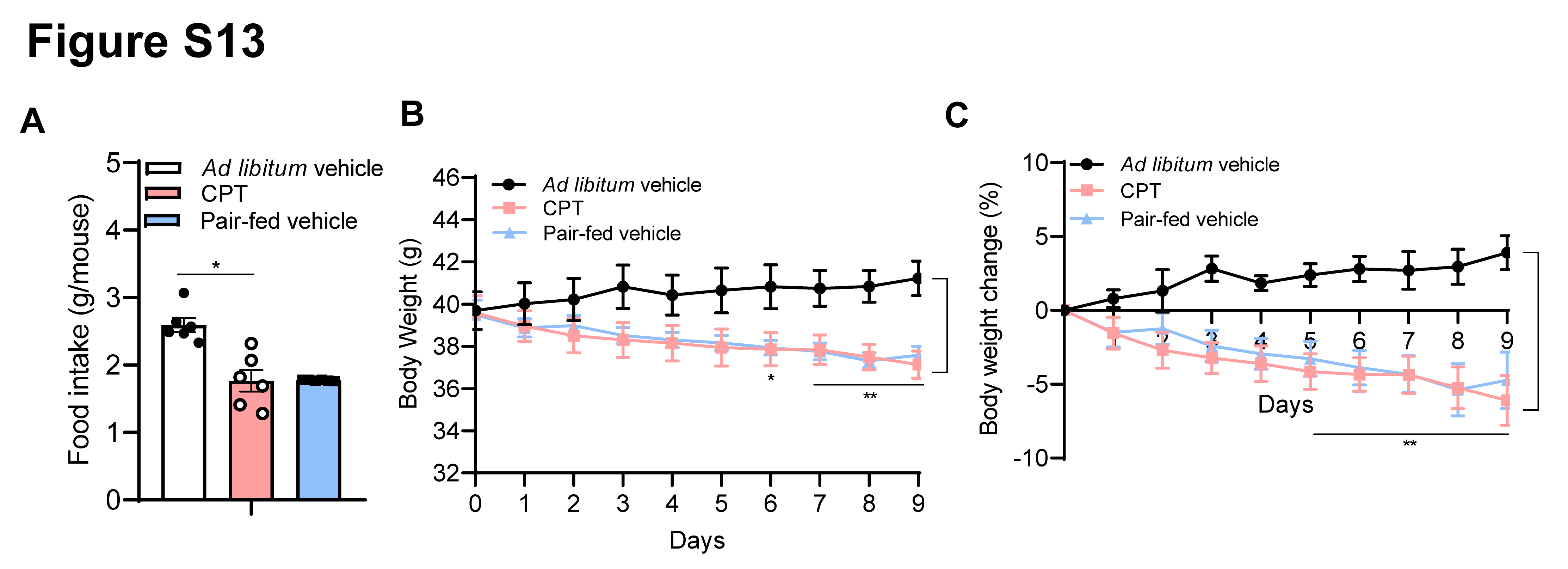

Supplement: S13 Fig — (A-C) Animal protocol 10: (A) The average of 9-day food intake (g day−1). The average daily food intake of the pair-fed vehicle groups was the same as for CPT-treated mice, an extra vehicle group were allowed ad libitum access to food. (B-C) Consecutive body weight and changes of body weight. Data are presented as mean ± SEM. n = 6 per group. The underlying data for this figure can be found in S1 Data. CPT, Camptothecin. (TIF) [file pbio.3001517.s013.tif]

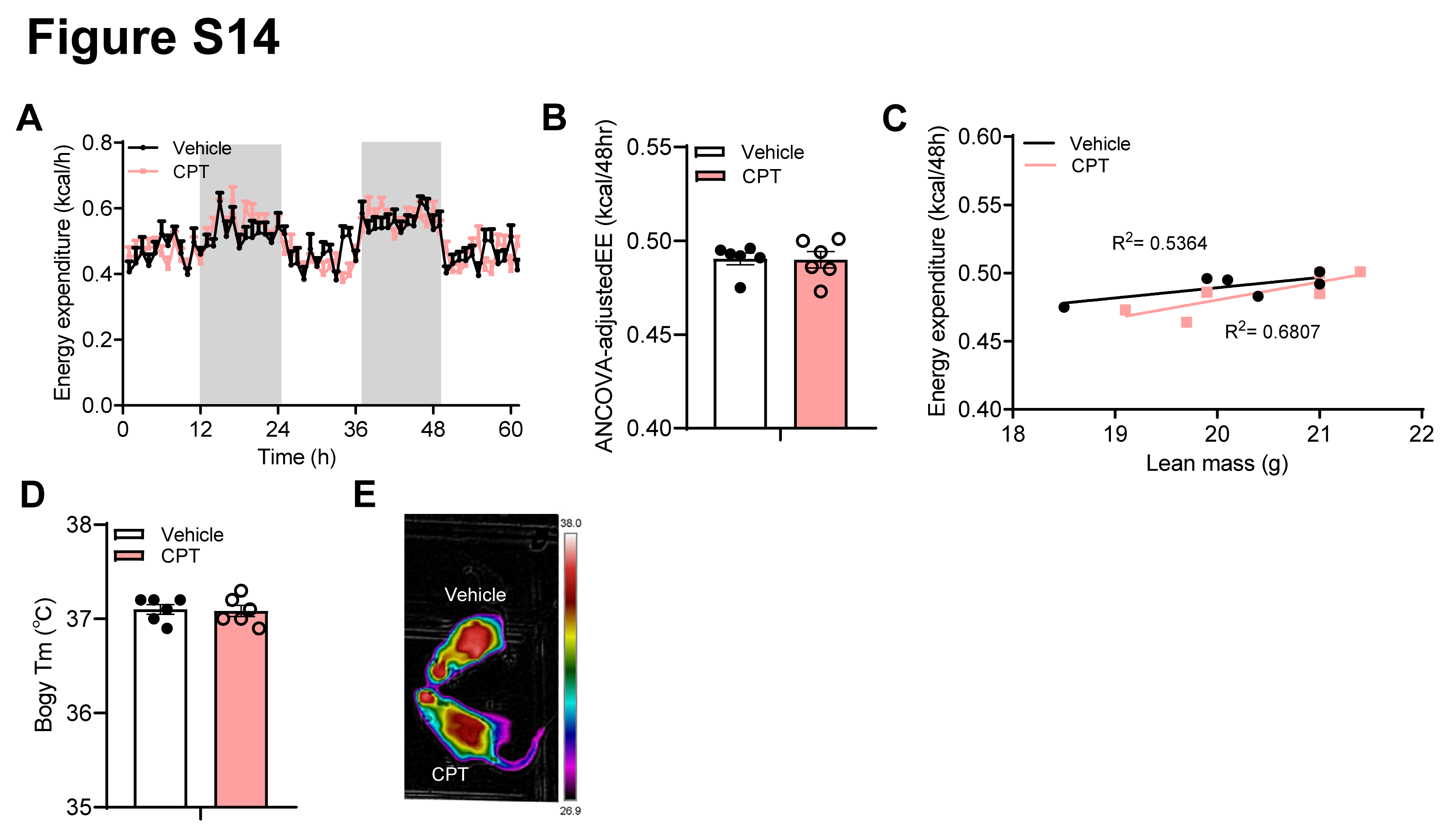

Supplement: S14 Fig — The EE of CPT-treated and vehicle control mice was shown in (A) uncorrected, (B) corrected for body mass, and (C) corrected for lean mass. (D) The body temperature of mice placed at room temperature (21°C) after 9 days of treatment. (E) Representative infrared images of mice. Images are captured using the rainbow high-contrast color palette in FLIR Research. n = 6 per group. The underlying data for this figure can be found in S1 Data. CPT, Camptothecin; DIO, diet-induced obese; EE, energy expenditure. (TIF) [file pbio.3001517.s014.tif]

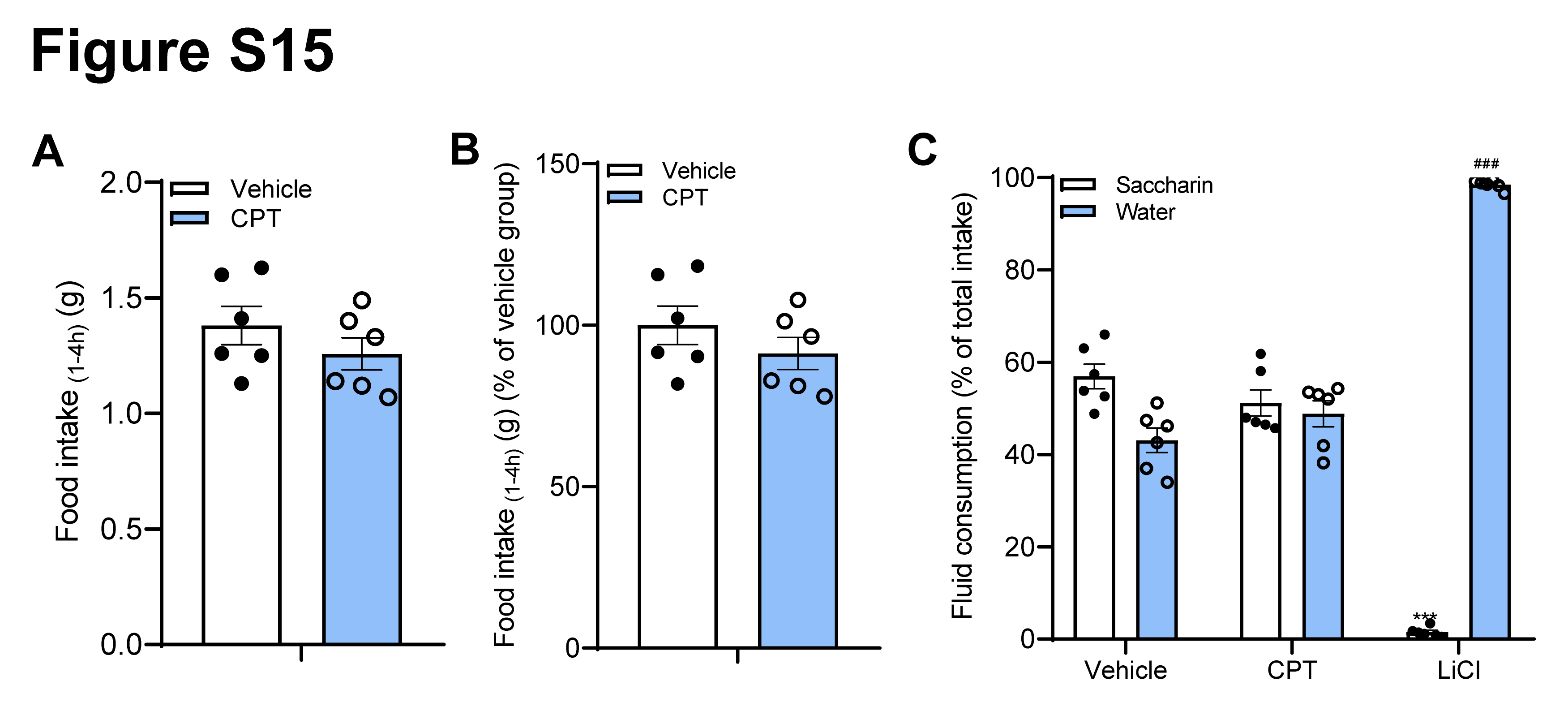

Supplement: S15 Fig — (A-C) Animal protocol 11: Cumulative food intake measured between 1 and 4 h post-CPT oral treatment as total grams (A) or percent (%) of vehicle control (B). (C) Saccharin and water consumption. Obese mice received either vehicle, CPT, or LiCl (positive control). Data are presented as mean ± SEM and analyzed using a two-way ANOVA with Bonferroni multiple comparison post-test to compare proportion of saccharin water and water consumption between groups of CPT or LiCl treatment to vehicle. *** (saccharin) or ### (water) P < 0.001. n = 6 per group. The underlying data for this figure can be found in S1 Data. CPT, Camptothecin; CTA, conditioned taste aversion. (TIF) [file pbio.3001517.s015.tif]

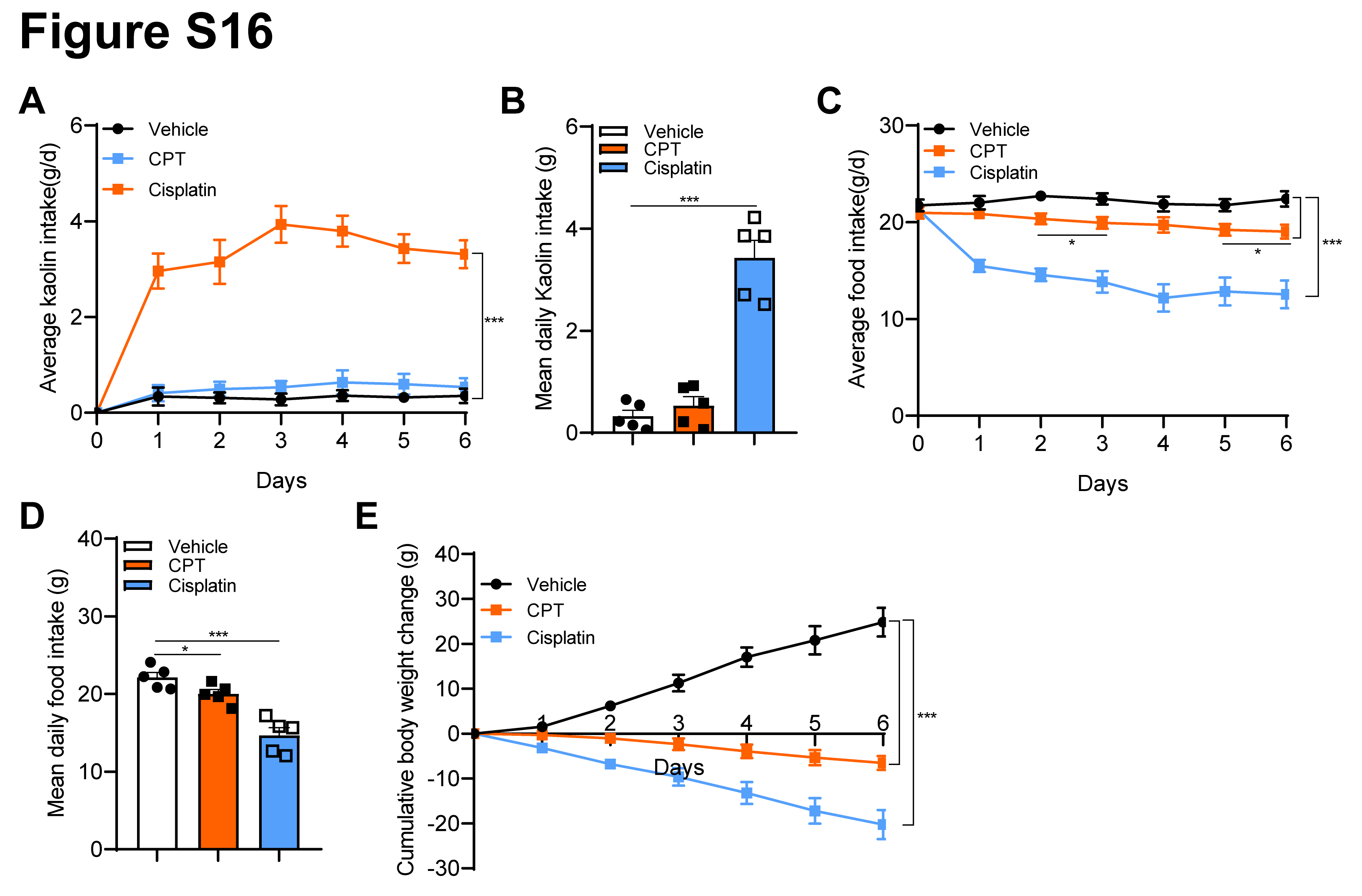

Supplement: S16 Fig — (A-E) Animal protocol 12: Rats were treated with vehicle, CPT (1 mg kg−1, gavage), or Cisplatin (6 mg kg−1, IP injection) for 6 days. (A) Kaolin intake and (B) the mean daily kaolin intake; (C) food intake and (D) the mean daily food consumption. (E) Cumulative change in body weight from baseline (g). Data are presented as mean ± SEM. * P < 0.05, ** P < 0.01, *** P < 0.001. n = 5 per group. The underlying data for this figure can be found in S1 Data. CPT, Camptothecin; IP, intraperitoneal. (TIF) [file pbio.3001517.s016.tif]

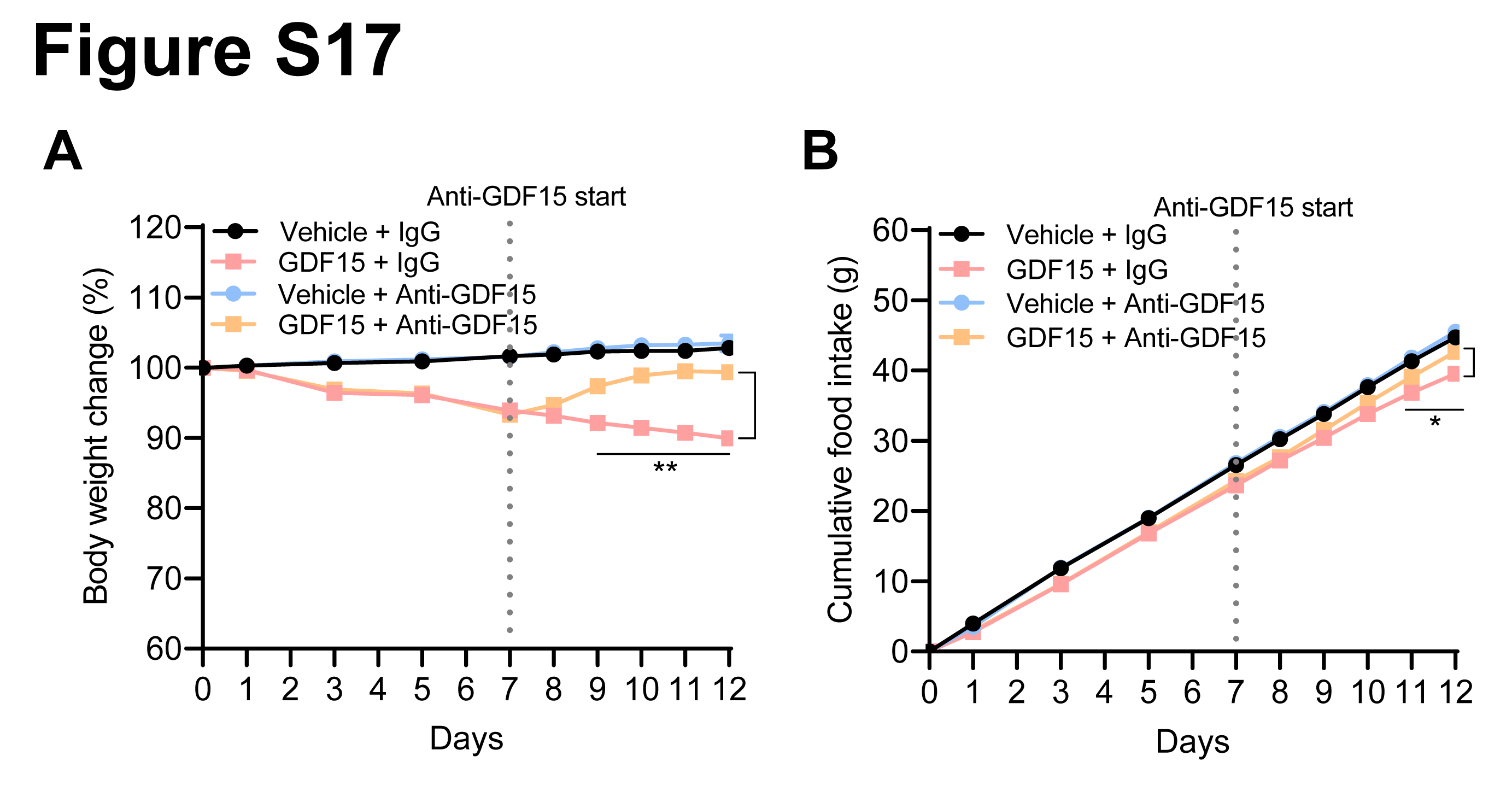

Supplement: S17 Fig — (A-B) Animal protocol 13: Three-month-old C57BL/6 mice were treated with either vehicle or GDF15 (0.1 mg kg−1) (IP injection, every other day) for 12 days. On day 7, the GDF15-treated mice were divided into 2 groups (n = 5 per group), receiving either GDF15 antibody (5 mg kg−1) or IgG control (IP injection, every other day). (A) Percentage change in body weight from baseline (%). (B) Cumulative food intake. Data are presented as mean ± SEM. The underlying data for this figure can be found in S1 Data. GDF15, growth differentiation factor 15; IgG, immunoglobulin G; IP, intraperitoneal. (TIF) [file pbio.3001517.s017.tif]

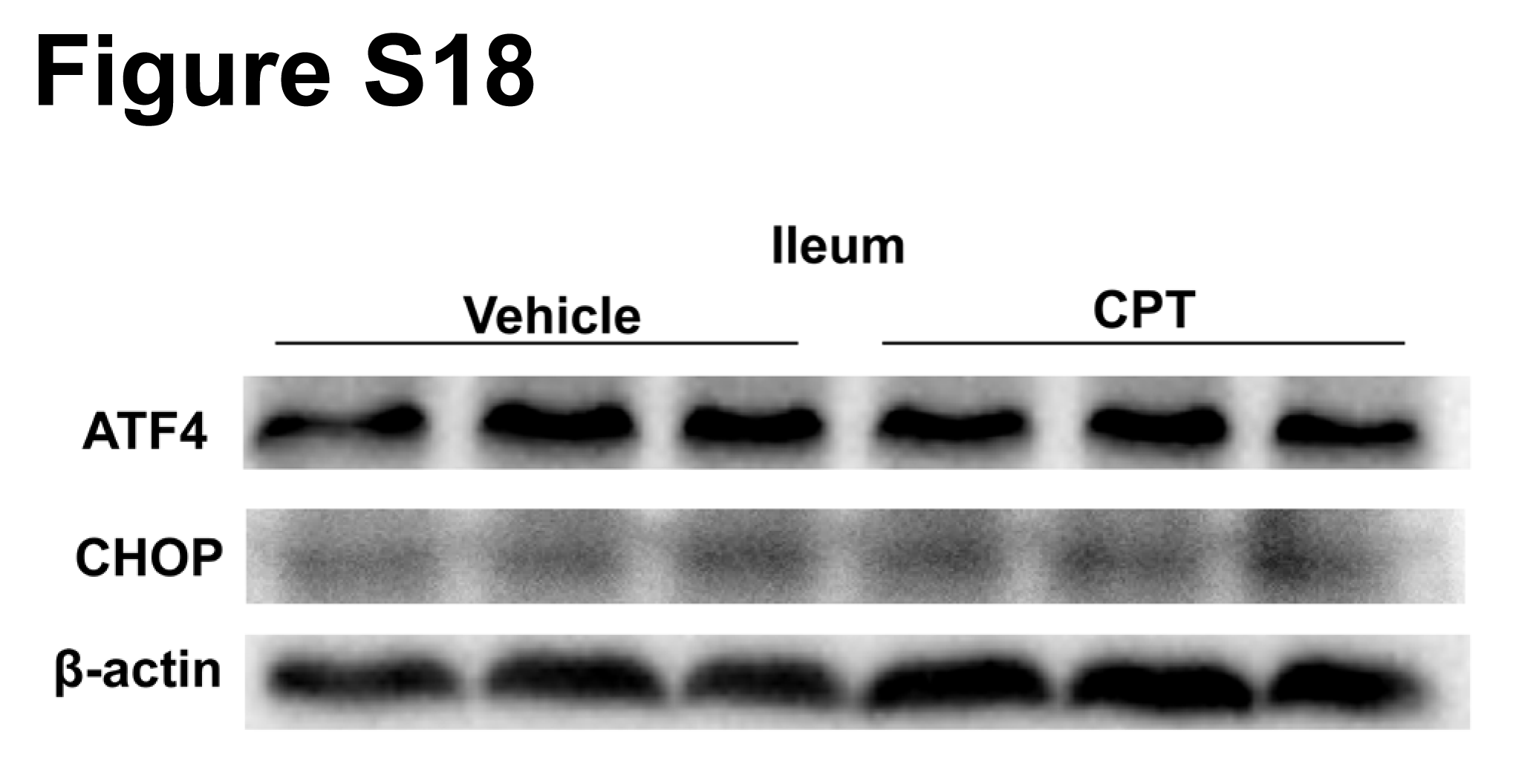

Supplement: S18 Fig — Immunoblot analysis of ATF4 and CHOP relative to β-actin in ileum of DIO mice treated with 1 mg kg−1 of CPT for 30 days (n = 3). The original blot for this figure can be found in S2 Raw Image. ATF4, activating transcription factor 4; CHOP, C/EBP homologous protein; CPT, Camptothecin; DIO, diet-induced obese; ISR, integrated stress response. (TIF) [file pbio.3001517.s018.tif]

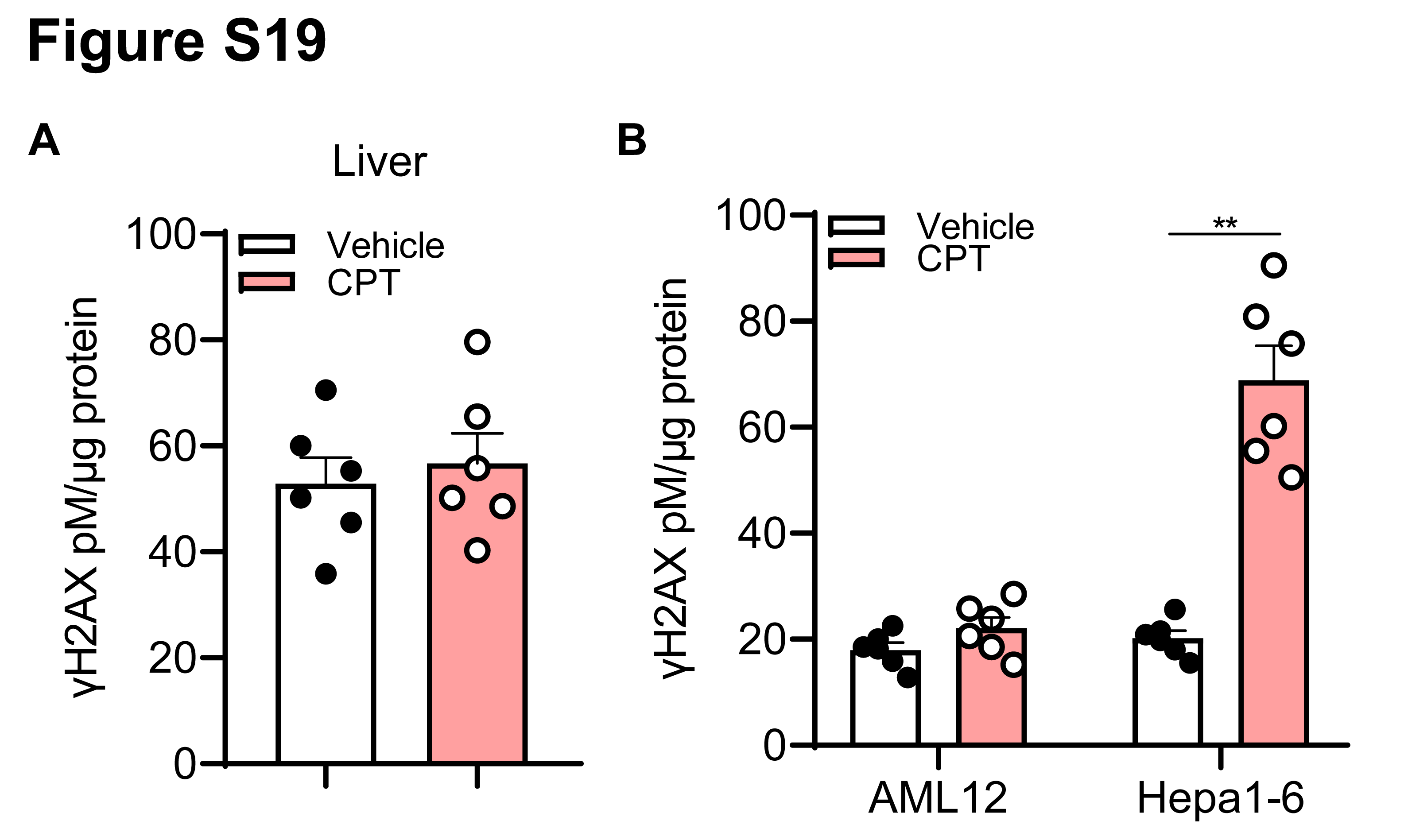

Supplement: S19 Fig — Levels of γH2AX in (A) liver of DIO mice treated with 1 mg kg−1 of CPT for 30 days, and (B) AML 12 cells and Hepa1-6 cells treated with 1 μM CPT for 24 h. Data are presented as mean ± SEM. n = 6 per group. The underlying data for this figure can be found in S1 Data. CPT, Camptothecin; DIO, diet-induced obese. (TIF) [file pbio.3001517.s019.tif]

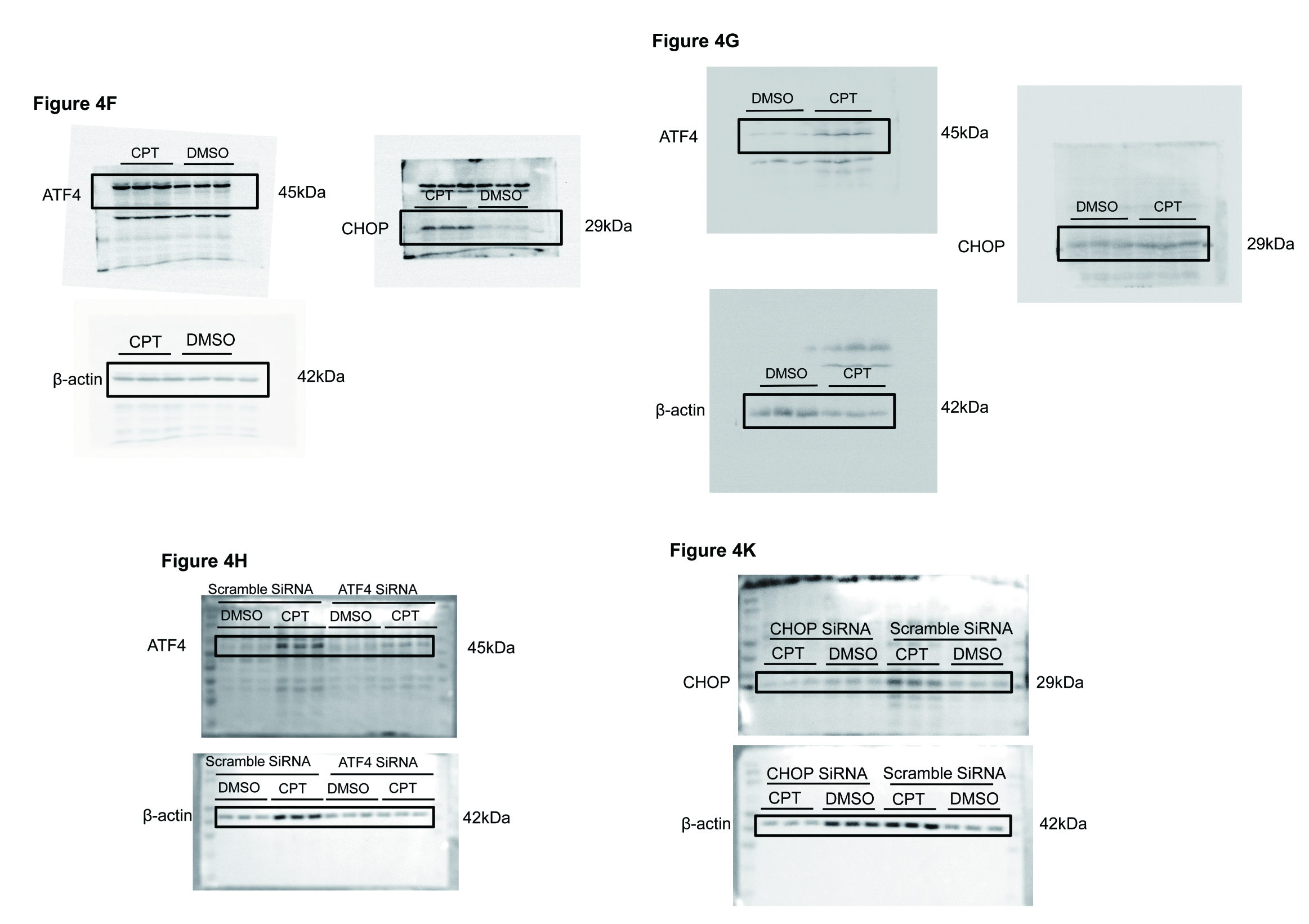

Supplement: S1 Raw Image — (TIF) [file pbio.3001517.s020.tif]

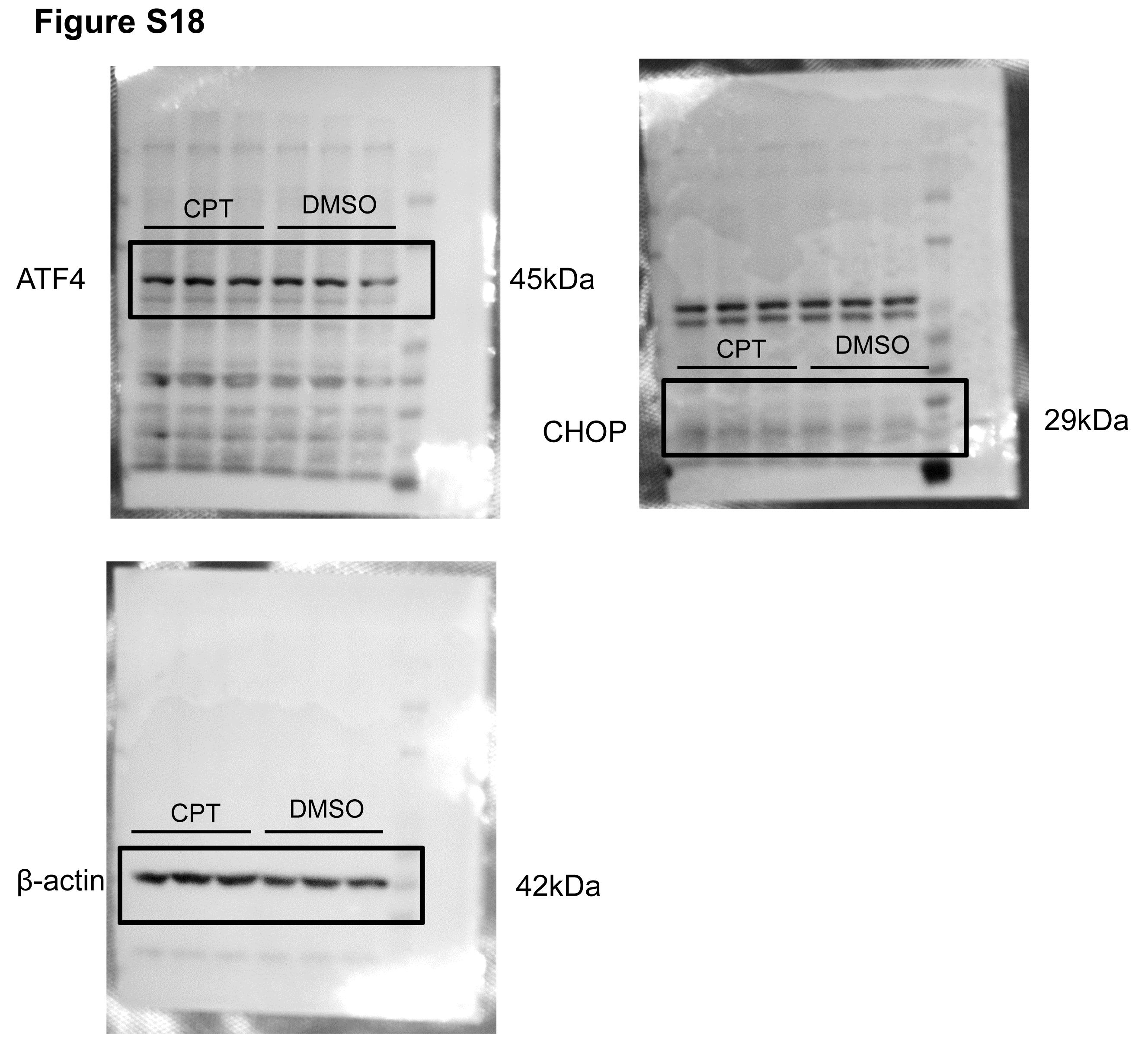

Supplement: S2 Raw Image — (TIF) [file pbio.3001517.s021.tif]
