# Supplementary material for: Camptothecin effectively treats obesity in mice through GDF15 induction
Source: PLoS Biol. 2022 Feb 24;20(2):e3001517. doi: 10.1371/journal.pbio.3001517 (PMC8870521; doi:10.1371/journal.pbio.3001517)
Supplement: S1 Table — (DOCX) [file pbio.3001517.s022.docx]

**S1 Table. Safety evaluation of CPT in DIO mice received either single acute or 30-day chronic treatment.**

| Parameters | Acute (single dose) | Chronic (30-Day) |
| --- | --- | --- |
| Diarrhea | Not observed | Not observed |
| Stomatitis | Not observed | Not observed |
| Cystitis | Not observed | Not observed |
| Dermatitis | Not observed | Not observed |
| Alopecia | Not observed | Not observed |
| White blood cell (10^8^ L^-1^) | 2.20 ± 0.16 | 2.22 ± 0.25 |
| Blood platelet (10^12^ L^-1^) | 1.32 ± 0.07 | 1.34 ± 0.11 |
| Hemoglobin (g dL^-1^) | 12.13 ± 0.31 | 11.53 ± 0.37 |
