# Supplementary material for: Camptothecin effectively treats obesity in mice through GDF15 induction
Source: PLoS Biol. 2022 Feb 24;20(2):e3001517. doi: 10.1371/journal.pbio.3001517 (PMC8870521; doi:10.1371/journal.pbio.3001517)
Supplement: S2 Table — (DOCX) [file pbio.3001517.s023.docx]

**S2 Table.** **List of shRNA oligonucleotides for gene knockdown.**

| shRNA oligonucleotides | Sequences (5′-3′) |
| --- | --- |
| *Scramble* shRNA | TTCTCCGAACGTGTCACGTAA |
| *Gdf15* shRNA | CCGGGTGTCACTGCAGACTTATGATCTCGAGATCATAAGTCTGCAGTGACACTTTTTG |
