# Supplementary material for: Camptothecin effectively treats obesity in mice through GDF15 induction
Source: PLoS Biol. 2022 Feb 24;20(2):e3001517. doi: 10.1371/journal.pbio.3001517 (PMC8870521; doi:10.1371/journal.pbio.3001517)
Supplement: S3 Table — (DOCX) [file pbio.3001517.s024.docx]

**S3 Table. Primers used for gene amplification.**

| Genes | Primer sequence (5′-3′) | Product (bp) | GenBank accession |
| --- | --- | --- | --- |
| *Gdf15* | F:TTTGGGGGGTGATGATGC  R:GCGACTTTCTGGGGAAACC | 130 | XM_021170582.2 |
| *Atf4* | F:GGGTTCTGTCTTCCACTCCA  R:AAGCAGCAGAGTCAGGCTTTC | 96 | NM_009716.3 |
| *Chop* | F:CCACCACACCTGAAAGCAGAA  R:AGGTGAAAGGCAGGGACTCA | 67 | NM_007837.4 |
| *β-actin* | F:GACCTGACTGACTACCTCAT  R:CGAAGTCAAGAGCCACATAG | 121 | NM_007393.5 |
